# Supplementary material for: Phase regulation enabling dense polymer-based composite electrolytes for solid-state lithium metal batteries
Source: Nat Commun. 2023 Oct 9;14:6296. doi: 10.1038/s41467-023-41808-3 (PMC10562402; doi:10.1038/s41467-023-41808-3)
Supplement: Supplementary file 1 — Supplementary Information [file 41467_2023_41808_MOESM1_ESM.pdf]

Supplementary Information

Phase regulation enabling dense polymer-based composite electrolytes for solid-state lithium metal batteries

Qian Wu[1,2], Mandi Fang[3], Shizhe Jiao[4], Siyuan Li[1,2], Shichao Zhang[1,2], Zeyu Shen[1,2], Shulan Mao[1,2], Jiale Mao[1,2], Jiahui Zhang[1,2], Yuanzhong Tan[5], Kang Shen[5], Jiaxing Lv[5], Wei Hu[4], Yi He[3,6] & Yingying Lu[1,2*]

[1] State Key Laboratory of Chemical Engineering, Institute of Pharmaceutical Engineering, College of Chemical and Biological Engineering, Zhejiang University, Hangzhou 310027, Zhejiang, China

[2] ZJU-Hangzhou Global Scientific and Technological Innovation Center, Zhejiang University, Hangzhou 311215, China

[3] College of Chemical and Biological Engineering, Zhejiang University, Hangzhou 310058, Zhejiang, China

[4] School of Future Technology, Department of Chemical Physics, and Anhui Center for Applied Mathematics, University of Science and Technology of China, Hefei 230026, China.

[5] Innovation Research Institute of Technology Center, Zhejiang Xinan Chemical Industrial Group Co. ltd, Hangzhou 311600, Zhejiang, China

[6] Department of Chemical Engineering, University of Washington, Seattle, Washington 98195, USA.

Correspondence and requests for materials should be addressed to Y.L. (email: yingyinglu@zju.edu.cn)

Supplementary Note 1

The nonuniform distribution of the DMF solvent and the porous structure of the PVDF electrolyte could always induce uneven Li deposition and rapid Li dendrite growth, as evidenced by previous reports[1, 2]. To further confirm this, we directly observed the Li deposition morphology in Li||Cu cells and tested the maximum Li deposition capacity in Li||Li cells. First, the plating morphologies of Li (1 mAh cm^{-2}) on Cu substrates were examined. As shown in Supplementary Fig. 10, the Li|PVDF|Cu cell present a highly loose and irregular deposition structure with many small Li particles, demonstrating the uneven Li deposition with the PVDF electrolyte. Second, to further quantitatively evaluate the effect of dense electrolyte structure on the uniform Li deposition, we assembled Li|PVDF|Li cell to test the critical deposition capacity (CDC), which is defined as the maximum endurable capacity of Li metal anode during cycling before Li dendrite growth and battery circuit short. The Li|PVDF|Li cell was cycled 5 times under current density of 0.1 mA cm^{-2} to form the SEI, which was then operated by continuously charge (one side Li metal stripping and the other side Li metal plating), until the battery presents a short circuit. As shown in Supplementary Fig. 11, the CDC in the PVDF electrolyte is approximately 1 mAh cm^{-2}. Given this, we can conclude that the uneven Li deposition and limited capacity always induce rapid Li dendrite growth and restrict the performance of the solid-state batteries under high loadings.

Supplementary Note 2

To evaluate the electronic conductivities of the formed SEI, we directly measured them following our previous reported method[3]. Since the SEI has similar properties with the solid-state electrolyte (SSE), the SEI could be considered as a thin SSE layer[4]. Therefore, the assembly of the device for investigating the electronic conductivity of the SEI can follow the Hebb-Wagner method for measuring the electronic conductivity of the SSE. Given this, we cycled the Li||NCM811 full cells for 20 times at 0.5C and 25°C and then disassembled them to obtain the SEI-coated Li metal anode (denoted as SEI-Li). The SEI-Li was clamped by two steel plates, and then the (-) Li/SEI/blocking electrode (+) cells were assembled. A dc voltage was applied in a chronoamperometric mode to the cells using an AMETEK. In the stationary state, the remaining current only stems from electrons diffusing through the SEI[5, 6]. The resistance of the SEI (R_e) could be calculated by equation (1):

$$R_e = \frac{\Delta V}{\Delta I_l} \quad (1)$$

where ΔV is the step voltage of 10 mV, and ΔI_l is the steady state response current. Thus, the electronic resistivity (ρ) of the SEI could be calculated by equation (2):

$$\rho = \frac{R_e \times A}{L} \quad (2)$$

where A is the area of SEI (2 cm^2), L is the thickness of SEI which has been obtained by cryogenic scanning transmission electron microscopy (cryo-STEM). Although the resistivity value calculated by this method may be a little lower than the actual value due to the underestimated A and the complicated structure of Li deposition, this does not hinder us from judging the electroconductivity of the SEI. As shown in Supplementary Fig. 49, the electronic resistivity of the SEI in PVMS-15 electrolyte is 4.23×10^5 Ω cm, almost equal to that in the PVDF electrolyte (4.21×10^5 Ω cm). The electronic conductivity is calculated to be 2.36×10^{-6} S cm^{-1} and 2.38×10^{-6} S cm^{-1}, respectively. This result demonstrates that the formation of metallic Mo and Mo-containing species show negligible influence on the electroconductivity of the SEI. In addition, the value of the electronic conductivities is acceptable for practical lithium metal batteries[7].

Supplementary Figures

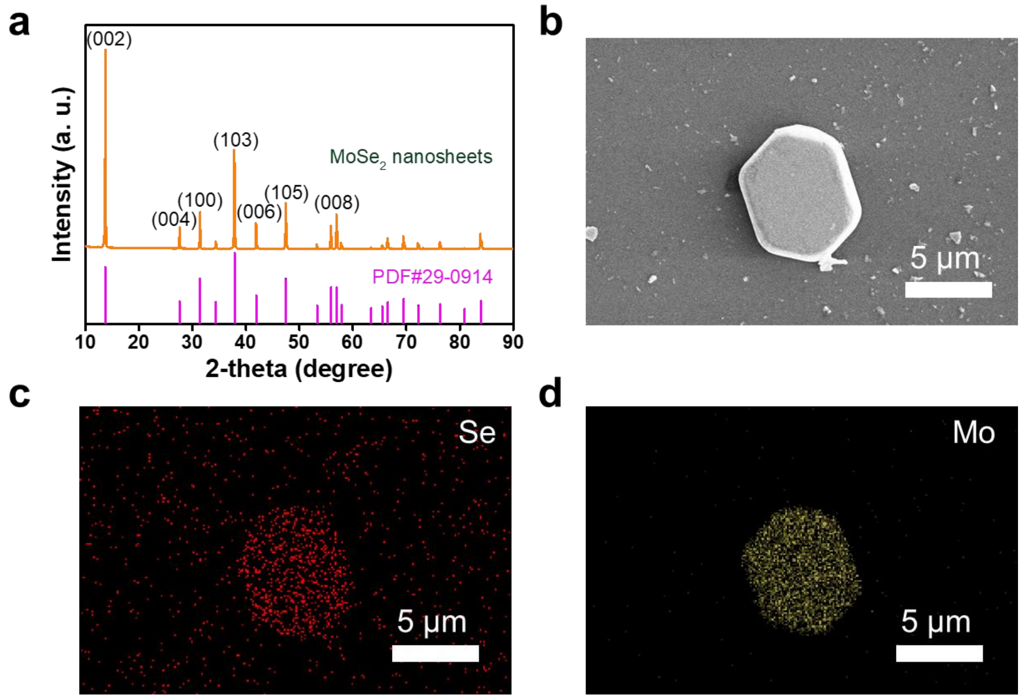

Supplementary Figure 1. Morphology and structure characterizations of MSs. a XRD pattern of $MoSe_2$ nanosheet. **b** SEM image of $MoSe_2$ nanosheet and its EDS mapping of Se (**c**) and Mo (**d**) element.

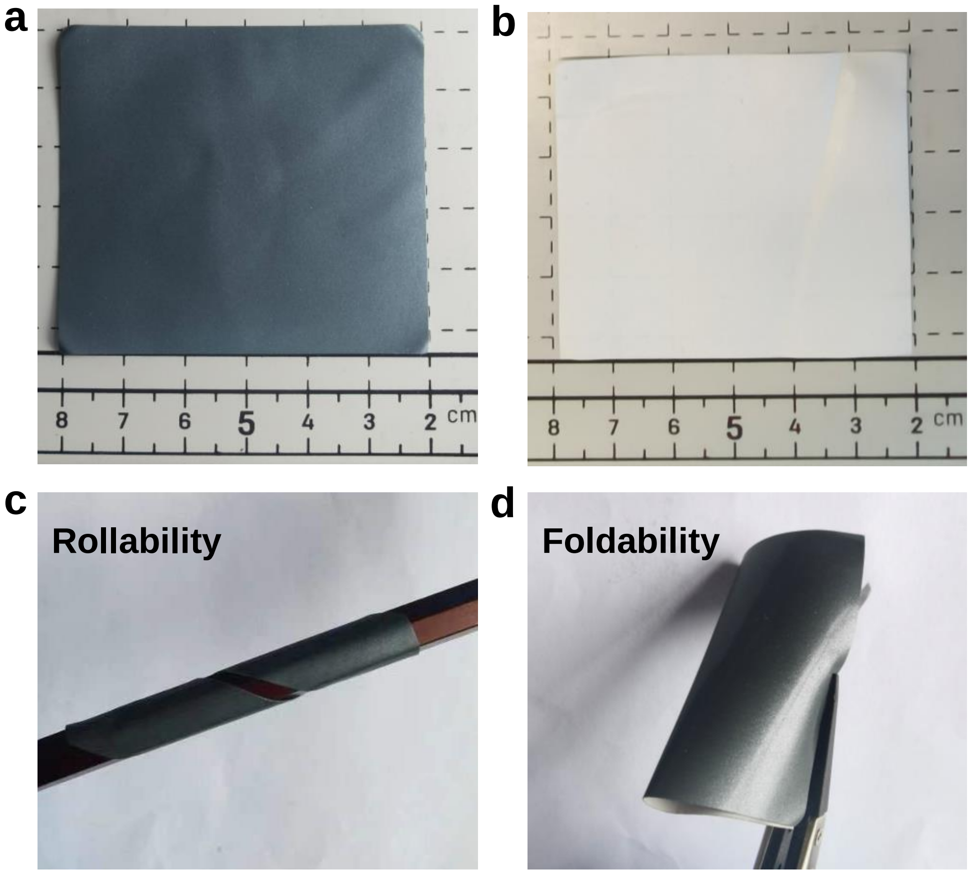

Supplementary Figure 2. Optical photographs of PVDF and PVMS-15 electrolytes. a PVDF electrolyte. **b** PVMS-15 electrolyte and rollability (**c**) and foldability (**d**) tests.

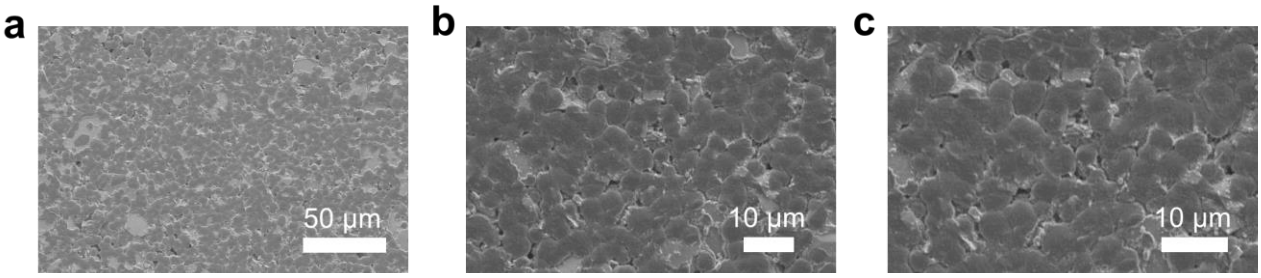

Supplementary Figure 3. SEM images of the PVMS-15 electrolyte. a 500×. **b** 1500×. **c** 2000×.

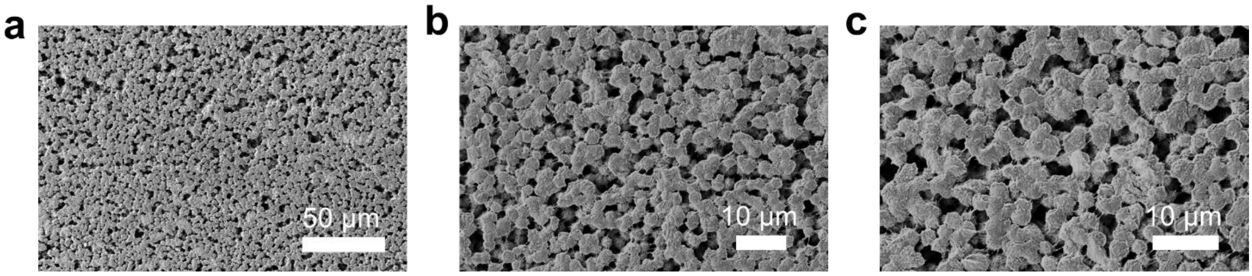

Supplementary Figure 4. SEM images of the PVDF electrolyte. a 500×. **b** 1500×. **c** 2000×.

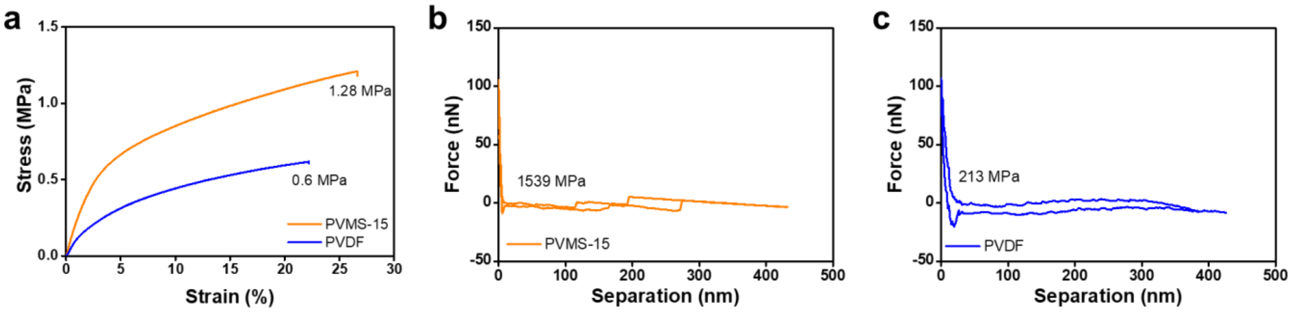

Supplementary Figure 5. Mechanical properties of electrolytes. a Tensile strength tested by stress-strain curves of PVDF and PVMS-15 electrolytes. Young’s modules tested by force-separation curves of PVMS-15 (**b**) and PVDF (**c**) electrolytes.

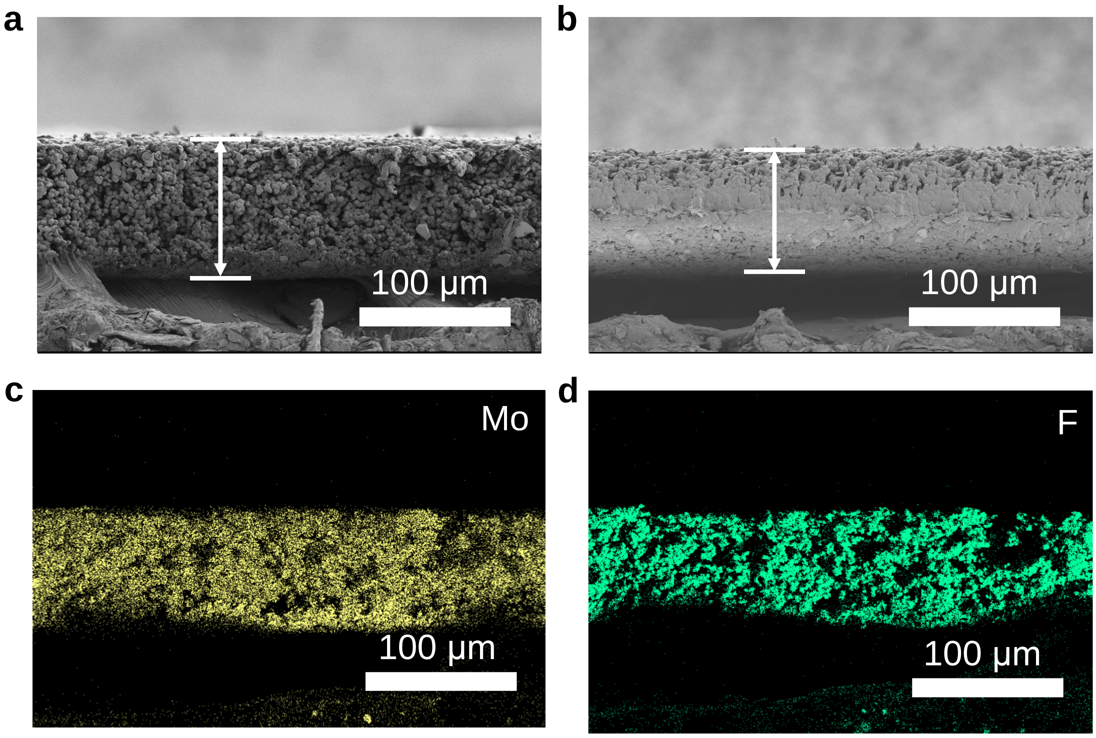

Supplementary Figure 6. Cross sectional SEM images of the electrolytes. a PVDF electrolyte. **b** PVMS-15 electrolyte and its EDS mappings of Mo (**c**) and F (**d**) elements**.**

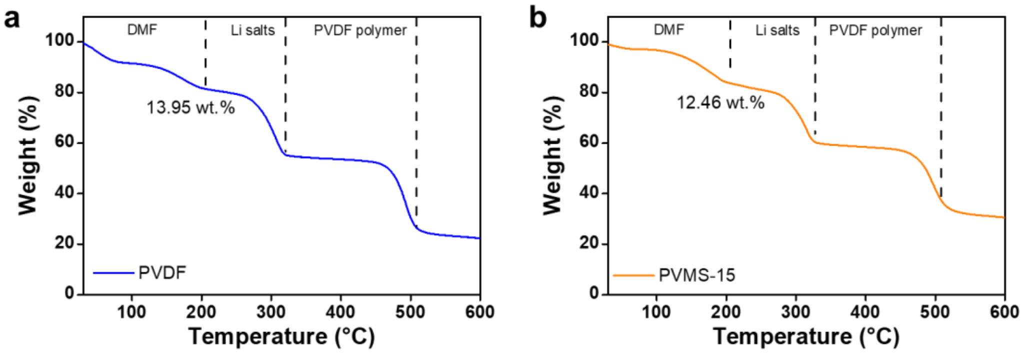

Supplementary Figure 7. Solvent content tested by TGA. TGA curves of PVDF (**a**) and PVMS-15 (**b**) electrolytes.

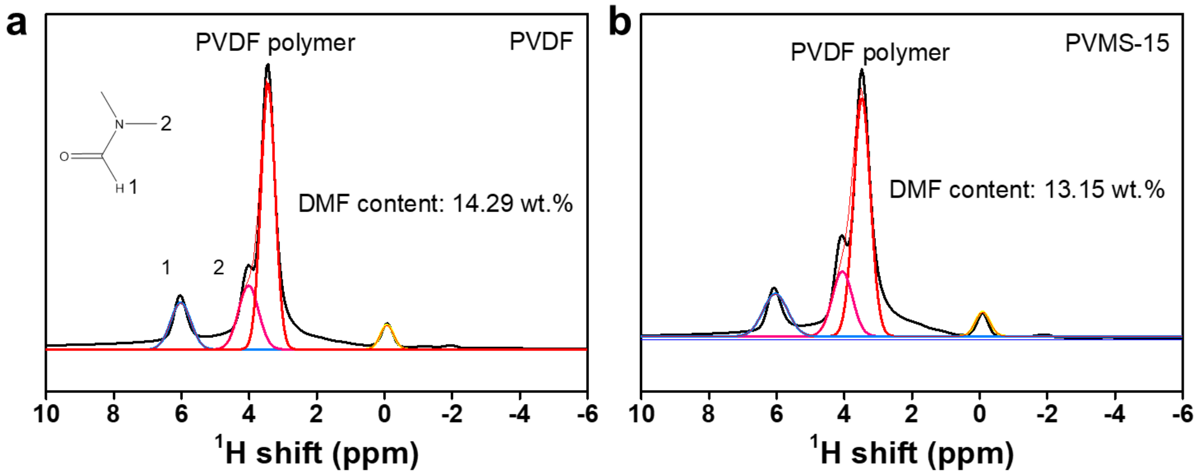

Supplementary Figure 8. Solvent content tested by ss-NMR. ^{1}H spectra of PVDF (**a**) and PVMS-15 (**b**) electrolytes.

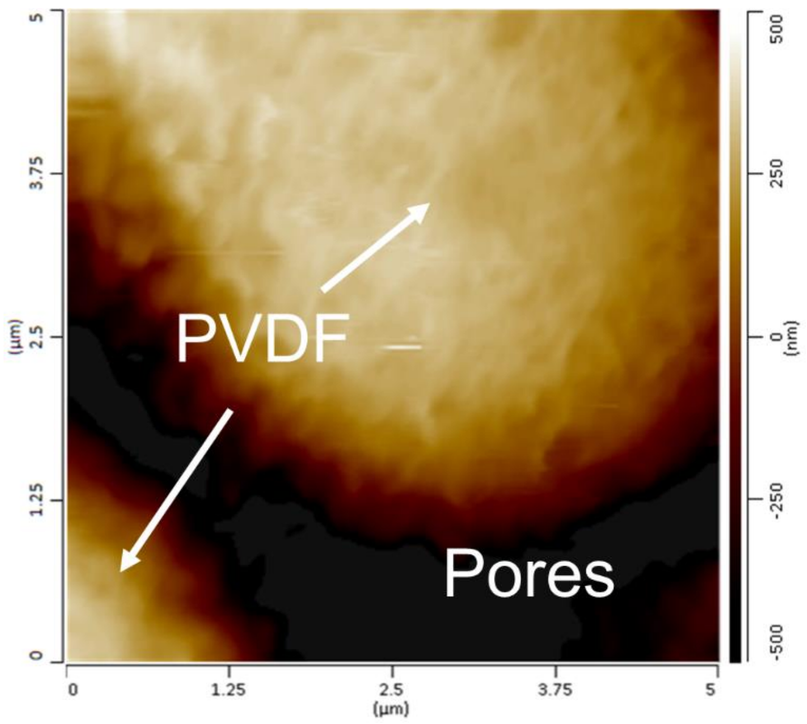

Supplementary Figure 9. AFM height map of the PVDF electrolyte.

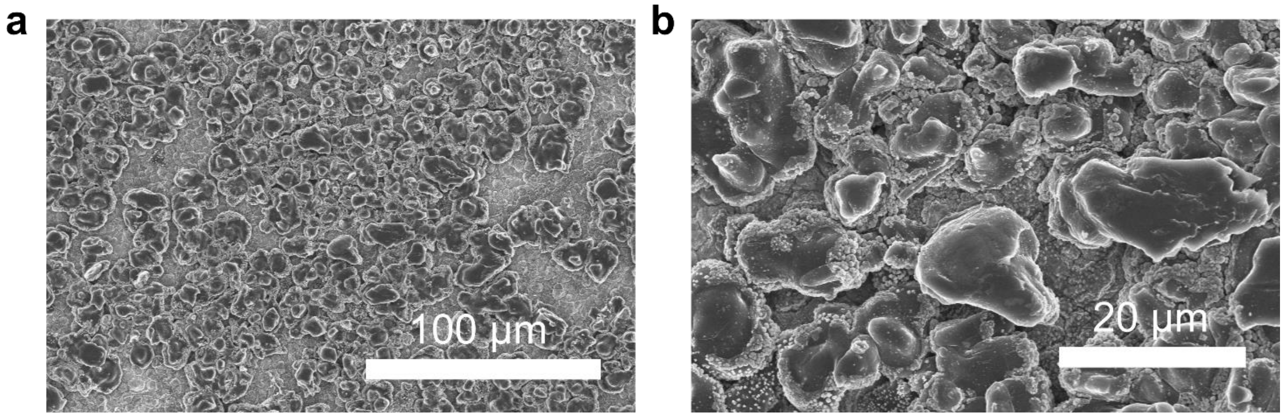

Supplementary Figure 10. SEM images of the Li deposition obtained by plating 1 mAh cm^{-2} Li on Cu substrate at 0.1 mA cm^{-2} in Li||Cu cells using PVDF electrolyte. a 500×. **b** 2000×.

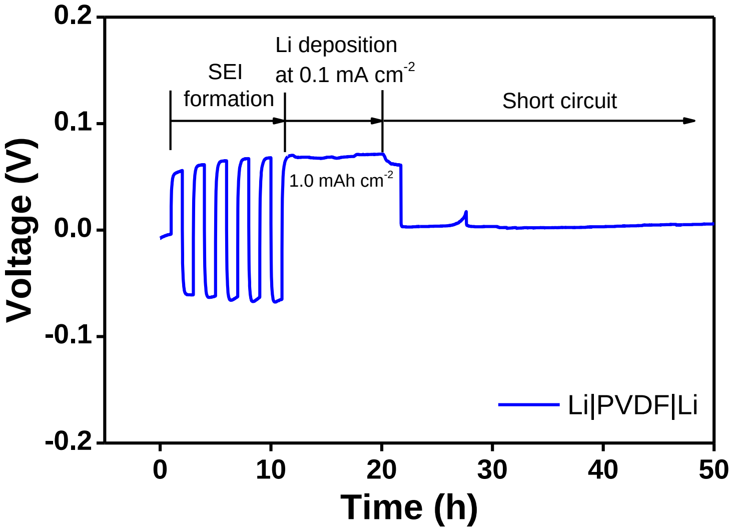

Supplementary Figure 11. CDC test curves in Li||Li cells at 0.1 mA cm^{-2} using PVDF electrolyte.

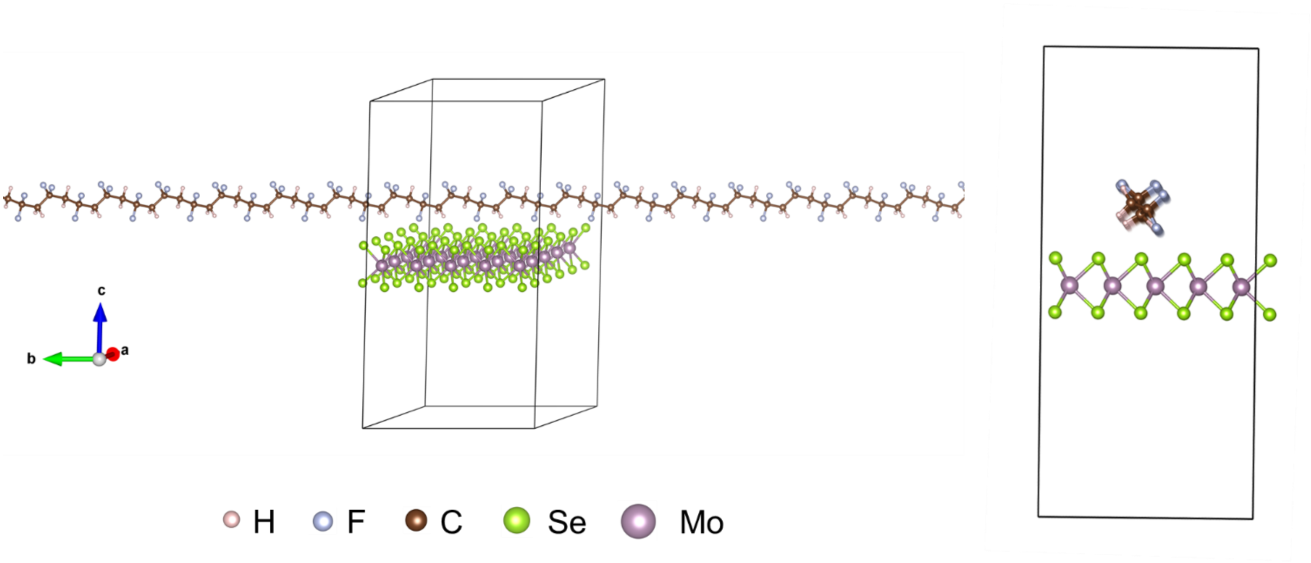

Supplementary Figure 12. DFT Geometry optimization of the interactions between MSs and PVDF at step 1.

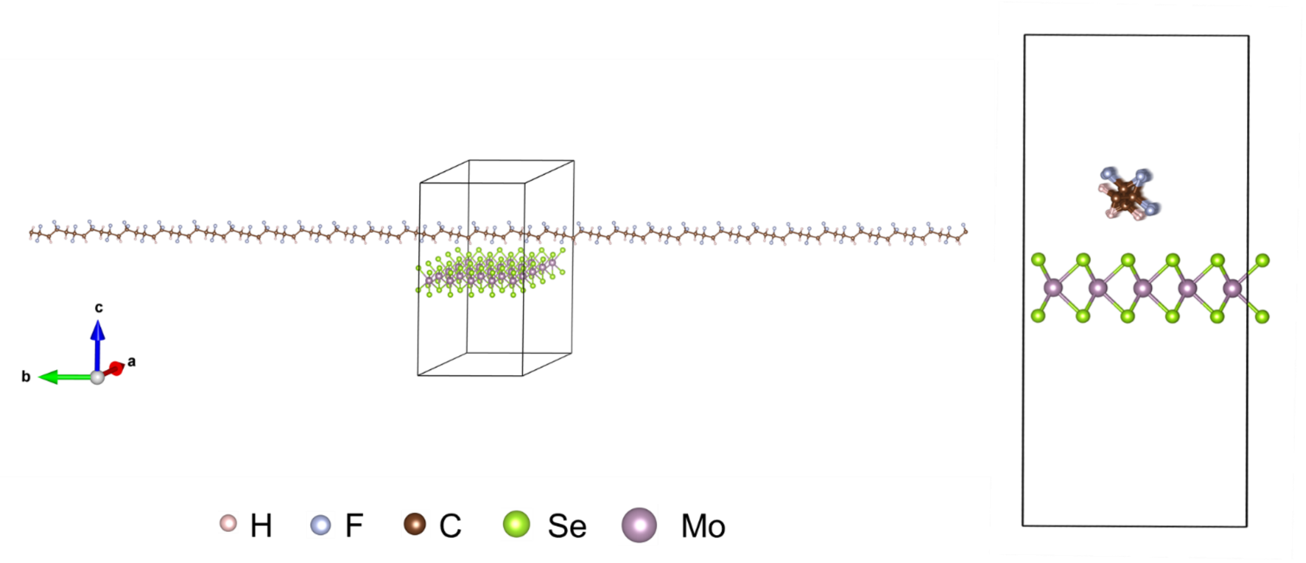

Supplementary Figure 13. DFT Geometry optimization of the interactions between MSs and PVDF at step 60.

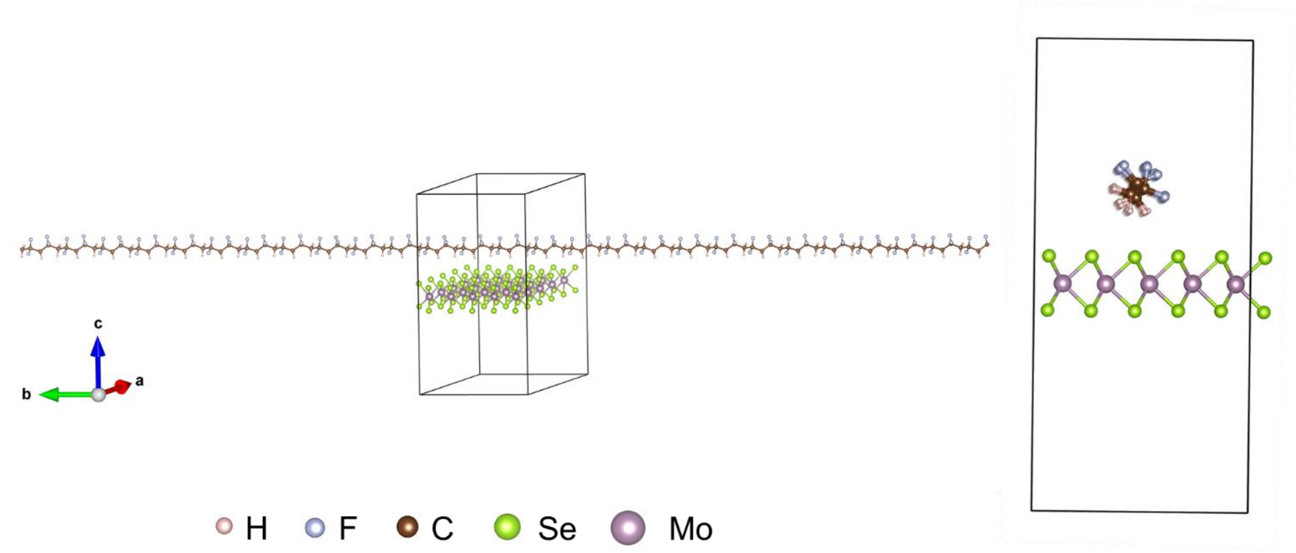

Supplementary Figure 14. DFT Geometry optimization of the interactions between MSs and PVDF at step 80.

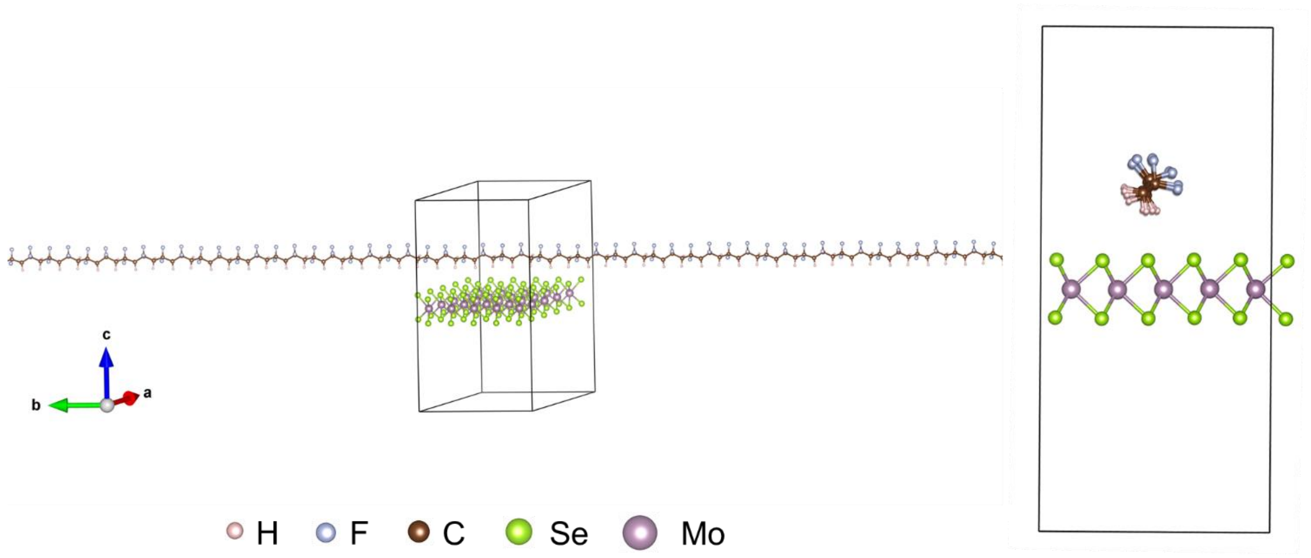

Supplementary Figure 15. DFT Geometry optimization of the interactions between MSs and PVDF at step 90.

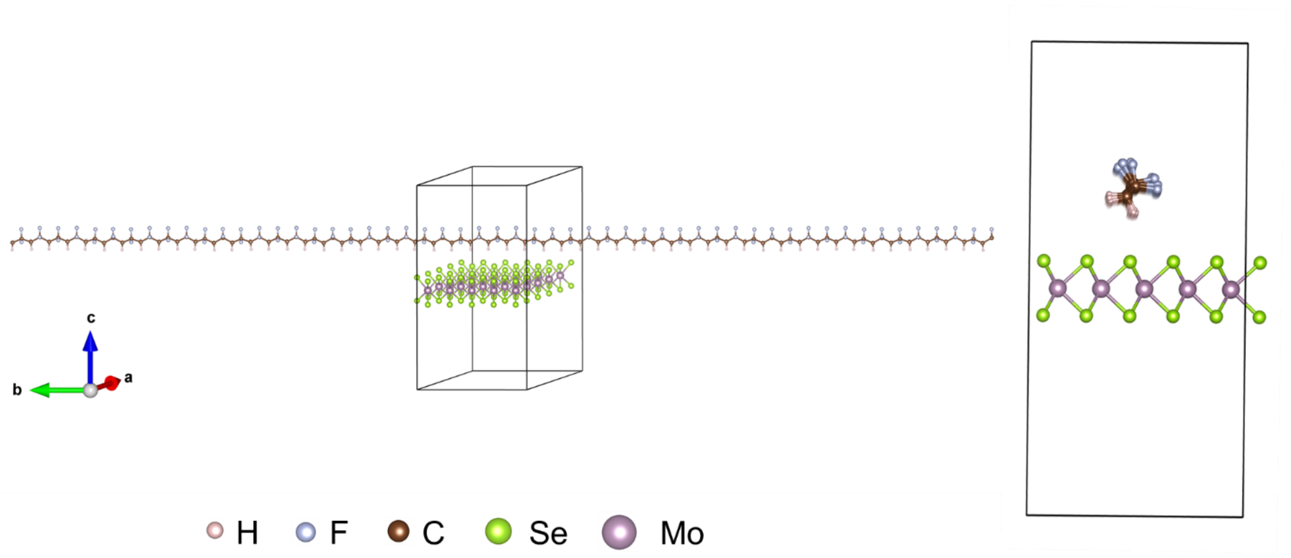

Supplementary Figure 16. DFT Geometry optimization of the interactions between MSs and PVDF at step 110.

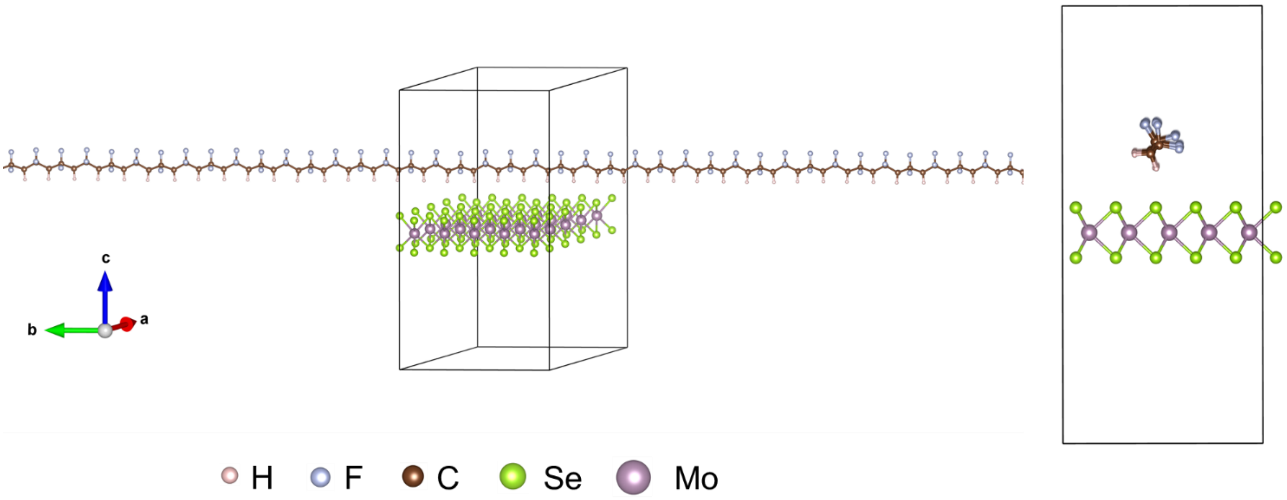

Supplementary Figure 17. DFT Geometry optimization of the interactions between MSs and PVDF at step 189.

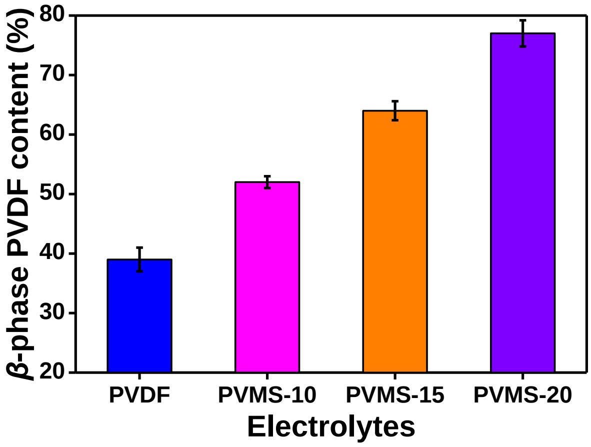

Supplementary Figure 18. Calculated *β*-phase PVDF content of the electrolytes from FTIR spectra. Values are means, and error bars were calculated by taking the standard errors from the measurements with three identical samples.

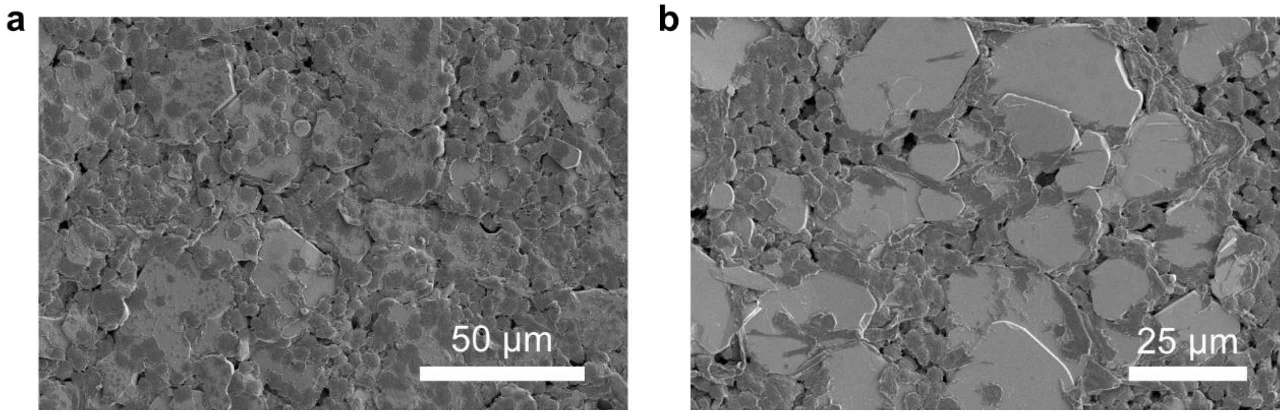

Supplementary Figure 19. SEM images of the PVMS-20 electrolyte at 700× (a) and 1000× (b).

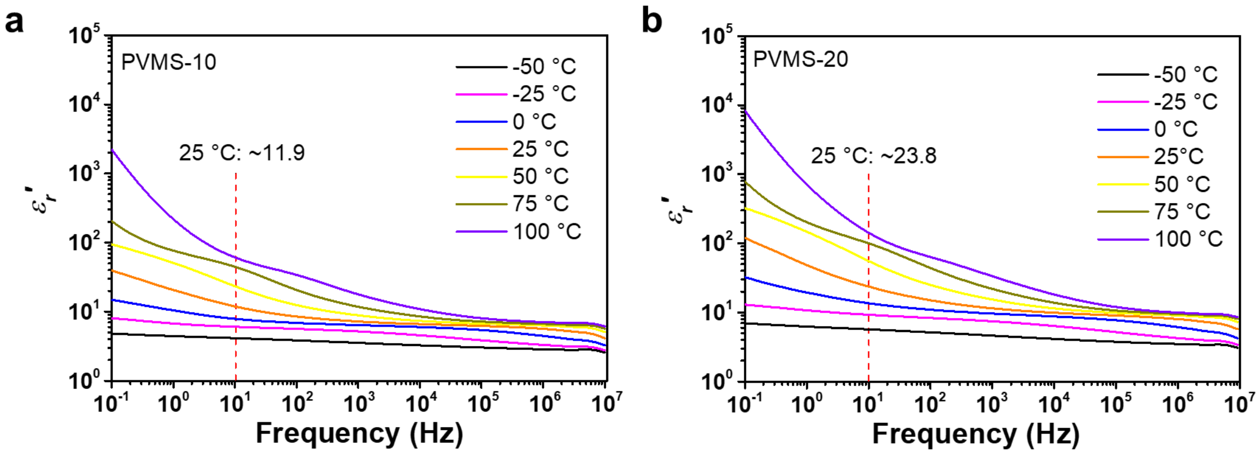

Supplementary Figure 20. Dielectric constant tests of the electrolytes. Real part (ε_r') of the relative permittivity as a function of frequency at different temperatures for the PVMS-10 (**a**) and PVMS-20 (**b**) electrolytes.

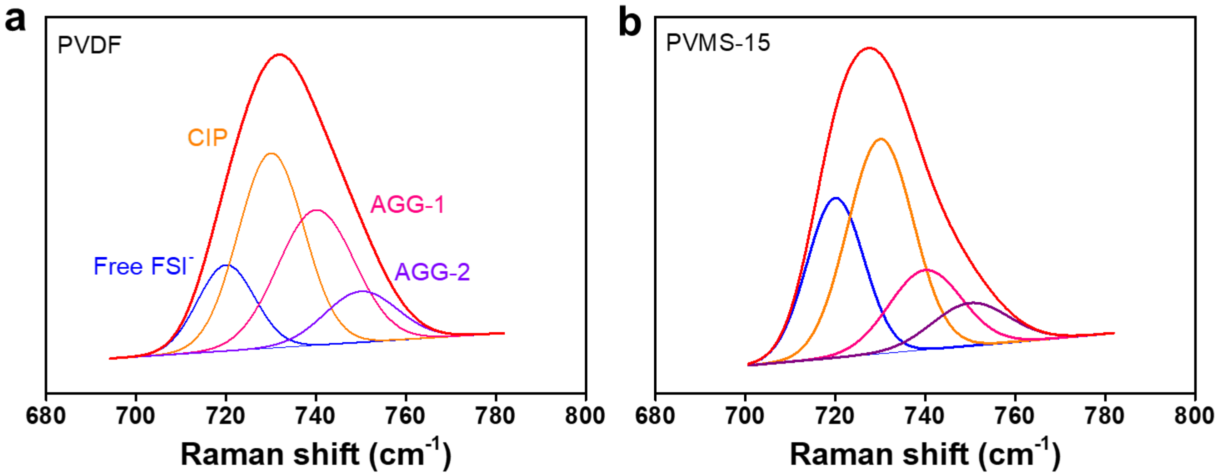

Supplementary Figure 21. Raman spectra of the electrolytes. a PVDF electrolyte. **b** PVMS-15 electrolyte.

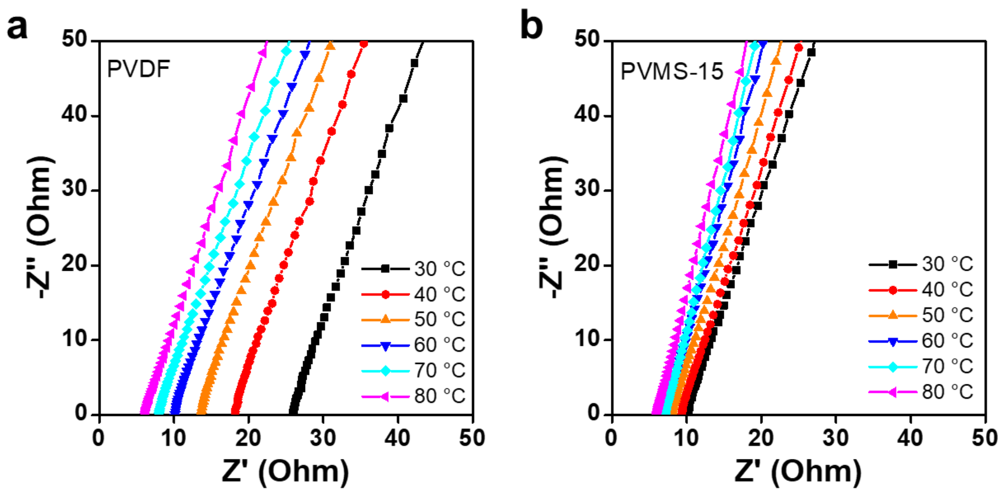

Supplementary Figure 22. Ionic conductivities tests of the electrolytes. EIS of SS||SS cells at different temperatures using the PVDF (**a**) and PVMS-15 (**b**) electrolytes.

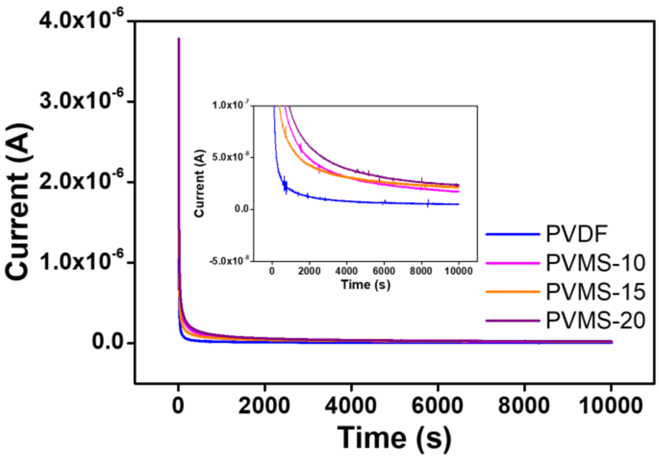

Supplementary Figure 23. Electronic conductivities tests of the electrolytes.

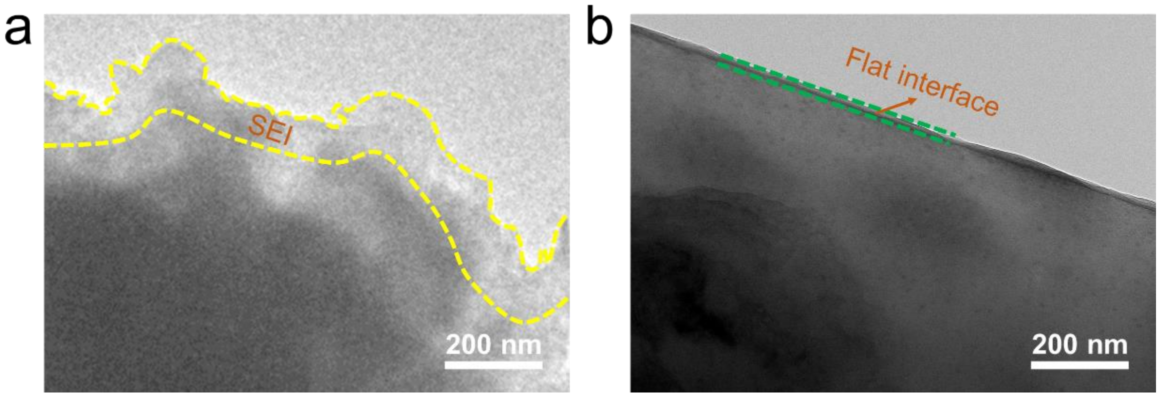

Supplementary Figure 24. SEI characterizations. Cryo-TEM images of formed SEI by PVDF (**a**) and PVMS-15 (**b**) electrolytes.

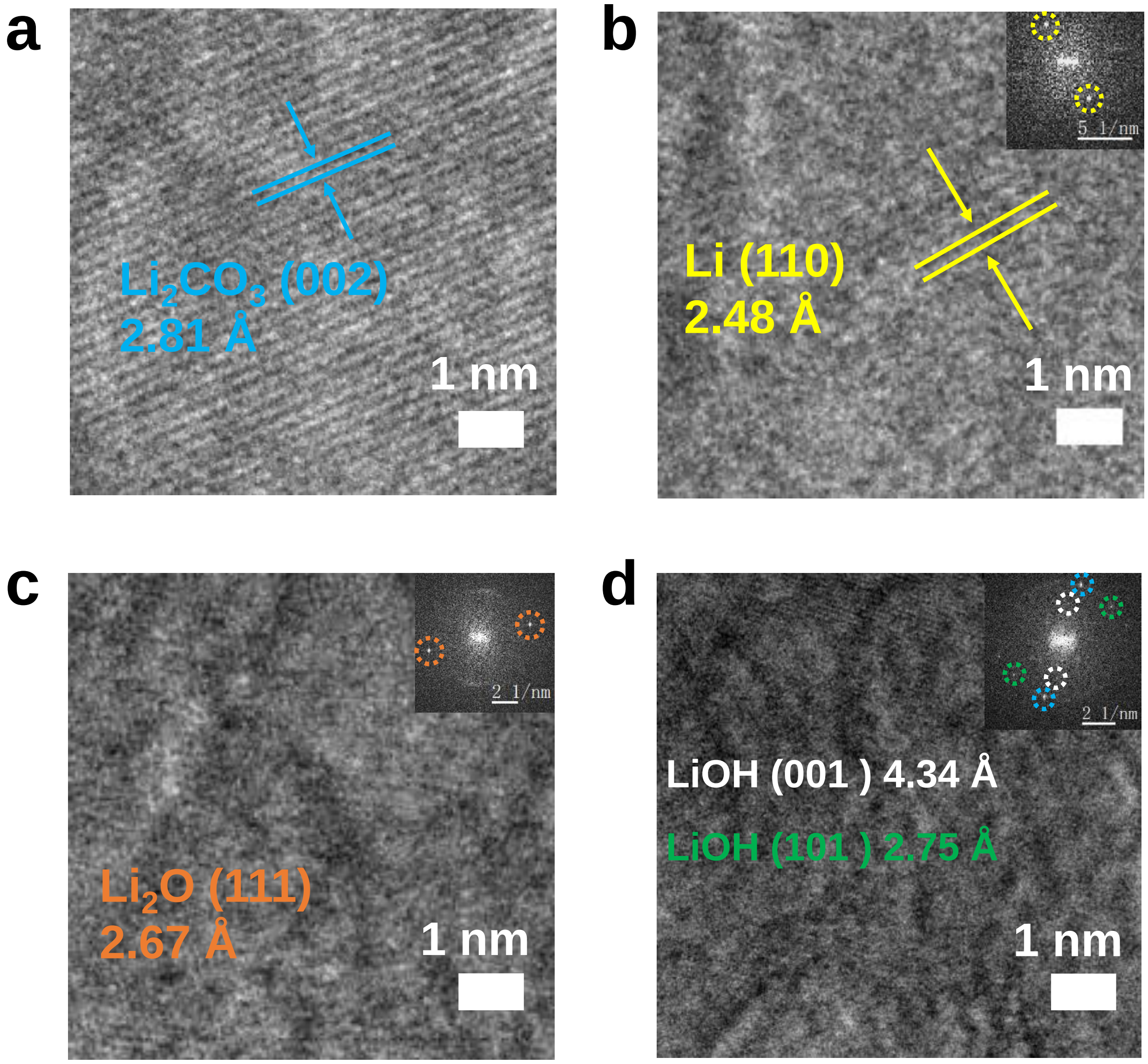

Supplementary Figure 25. Nanostructures of the SEI formed by PVMS-15 electrolyte. HRTEM images of Li_2CO_3 (**a**), Li (**b**), Li_2O (**c**) and LiOH (**d**) crystals.

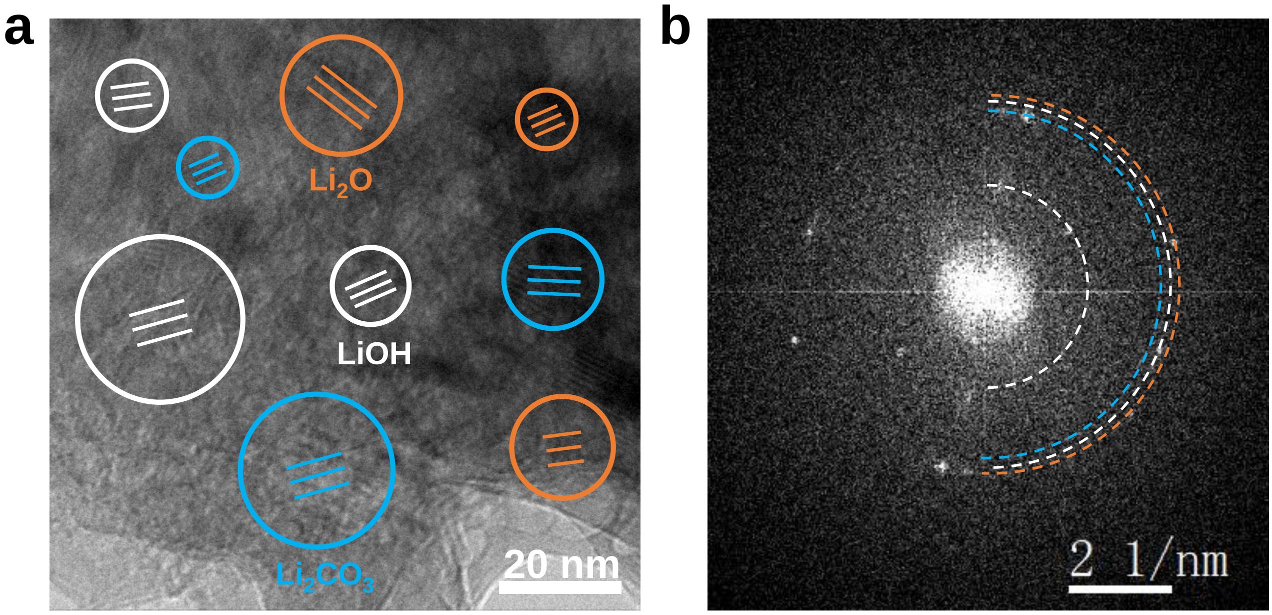

Supplementary Figure 26. SEI images formed by PVDF electrolyte. a Enlarged TEM image of SEI formed by PVDF electrolyte. **b** FFT pattern.

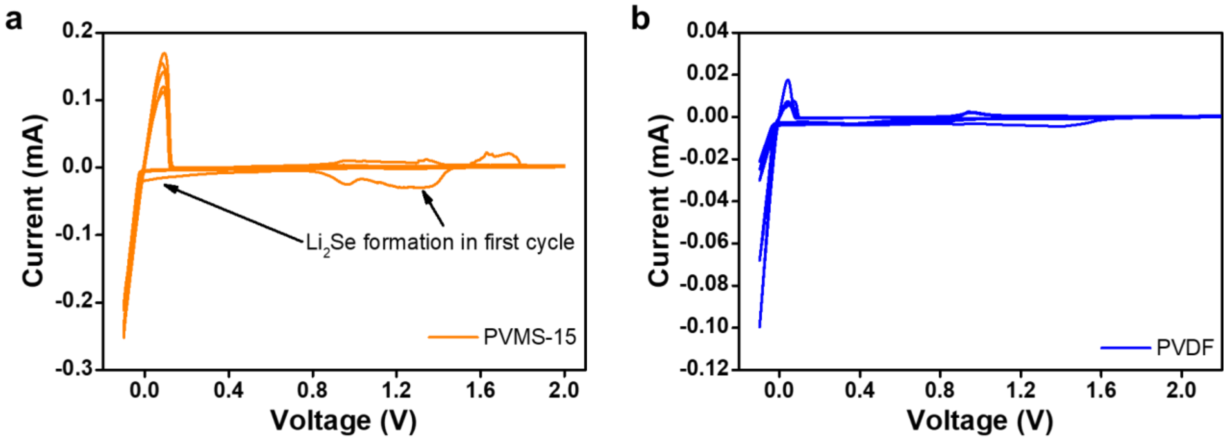

Supplementary Figure 27. Li metal compatibility tests of the electrolytes. CV curves of Li||Cu cells using PVMS-15 (**a**) and PVDF (**b**) electrolytes.

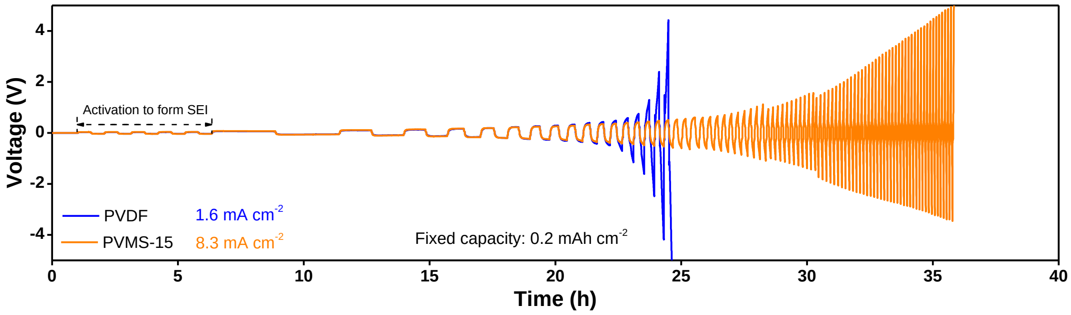

Supplementary Figure 28. CCD tests of the electrolytes using capacity control method.

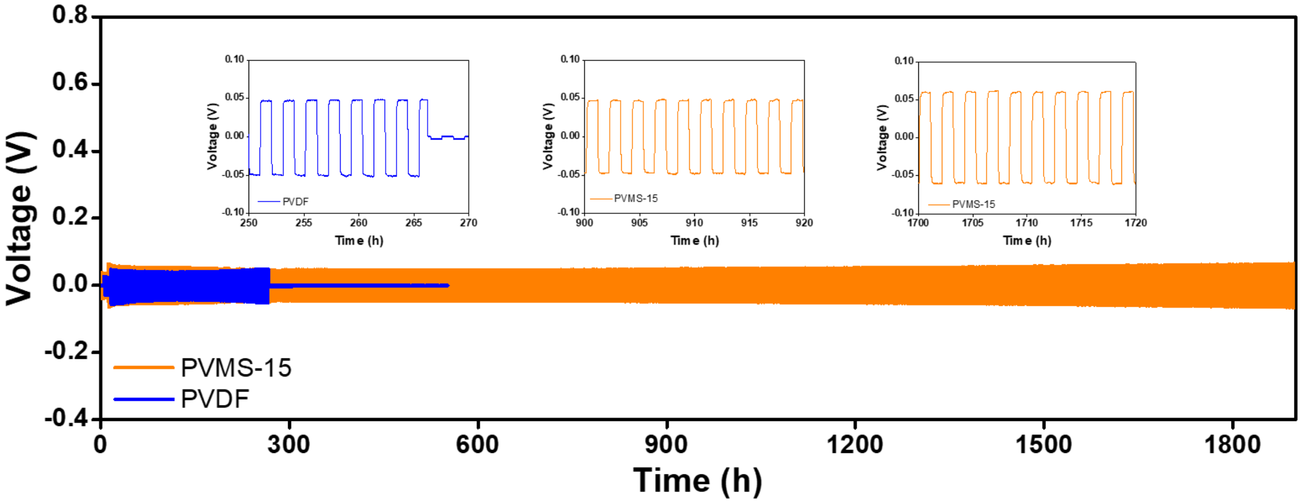

Supplementary Figure 29. Galvanostatic cycling curves of Li||Li cells with PVDF and PVMS-15 electrolytes at current density of 0.1 mA cm^{-2}.

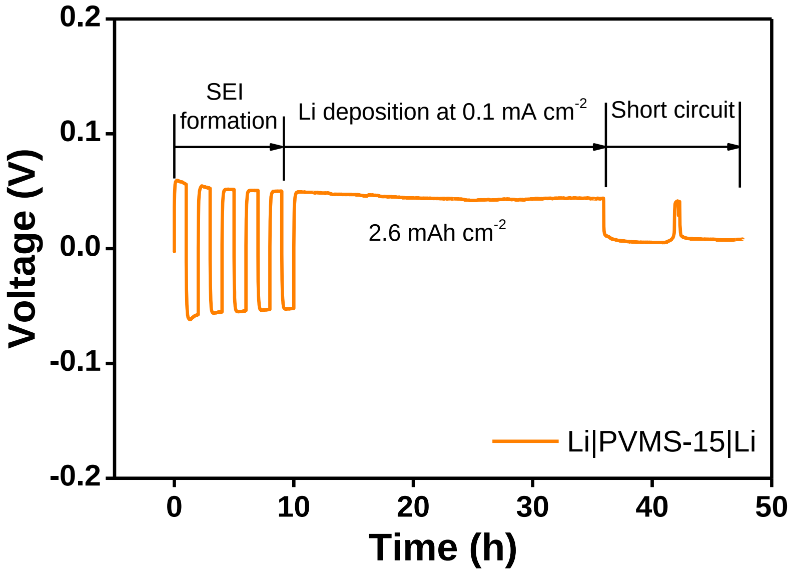

Supplementary Figure 30. CDC test curves in Li||Li cells at 0.1 mA cm^{-2} using PVMS-15 electrolyte.

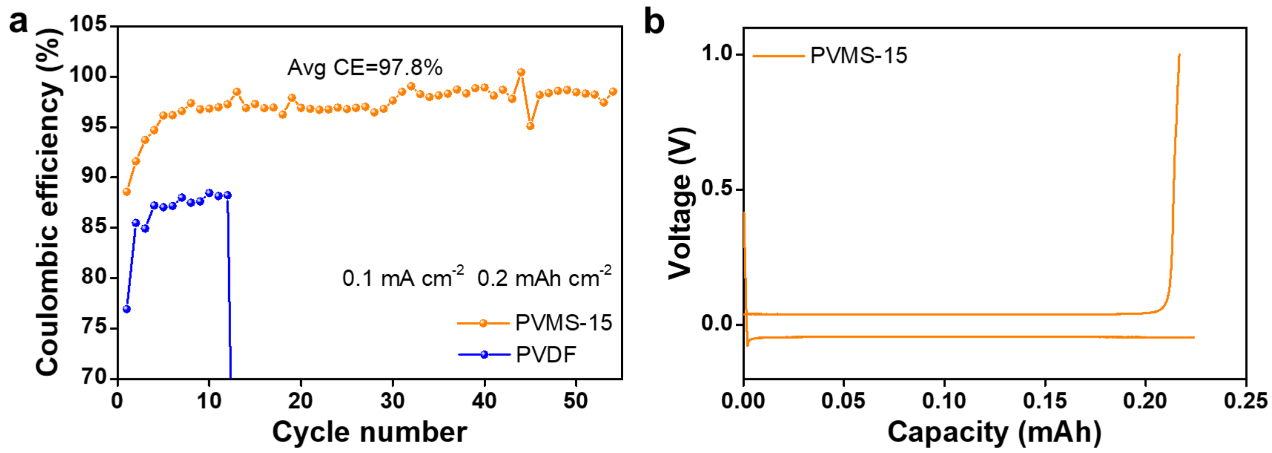

Supplementary Figure 31. CE tests in Li||Cu cells using PVDF and PVMS-15 electrolytes. a Long-term cycling stability. **b** Li deposition-stripping curve in the 30th cycle.

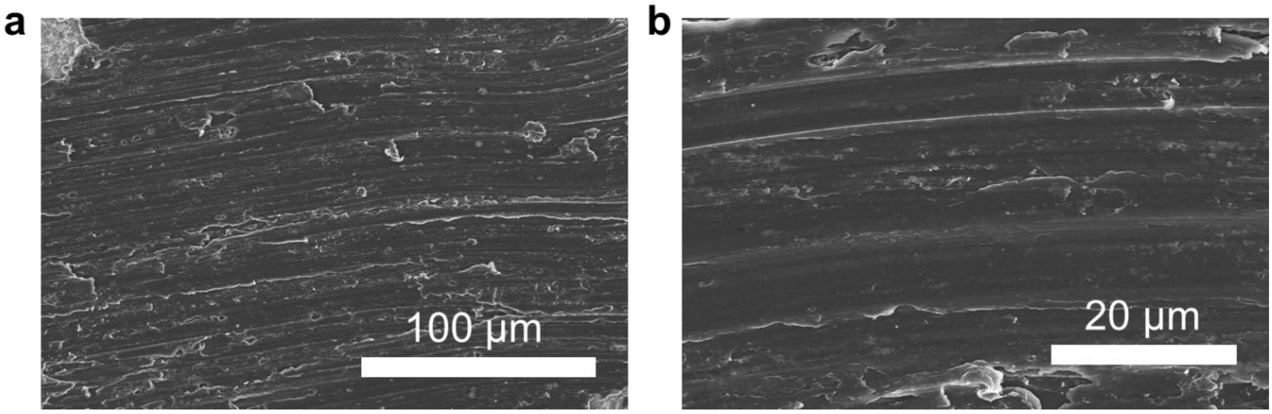

Supplementary Figure 32. SEM images of the Li deposition obtained by plating 1 mAh cm^{-2} Li on Cu substrate at 0.1 mA cm^{-2} in Li||Cu cells using PVMS-15 electrolyte. a 500×. **b** 2000×.

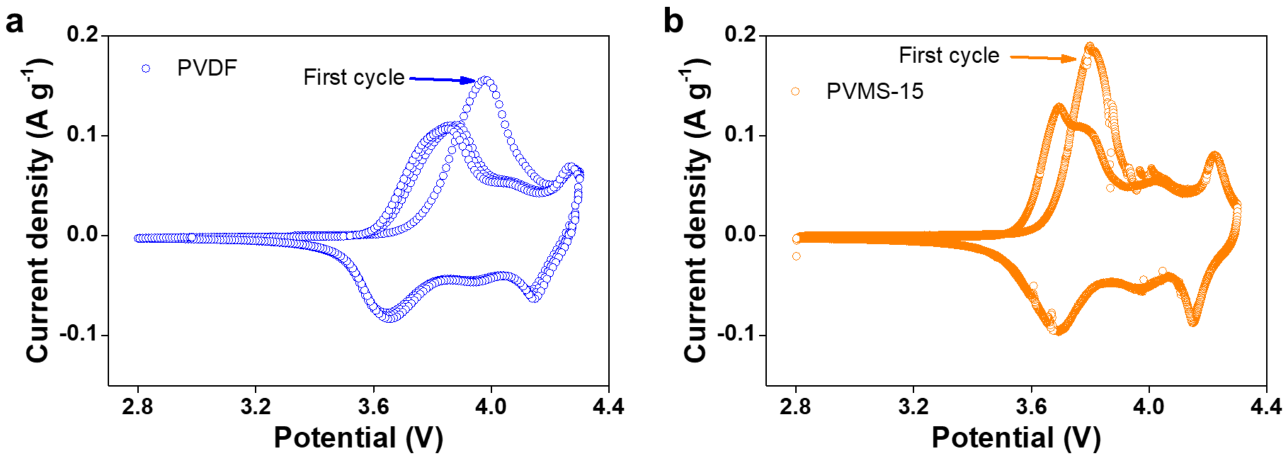

Supplementary Figure 33. CV tests of Li||NCM811 full cells. CV curves of Li|PVDF|NCM811 (**a**) and Li|PVMS-15|NCM811 (**b**) cells in voltage range of 2.8~4.3 V at scanning rate of 0.05 mV s^{-1}.

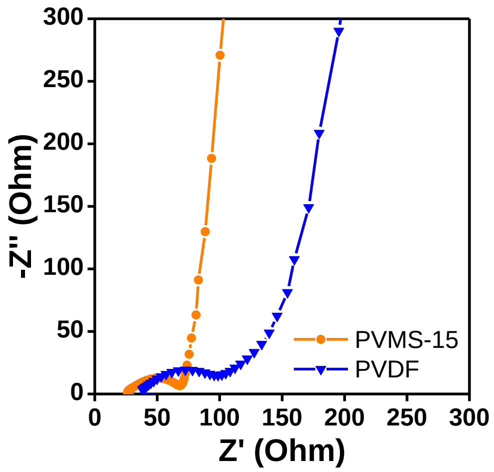

Supplementary Figure 34. EIS of Li|PVDF|NCM811 and Li|PVMS-15|NCM811 cells before rate tests.

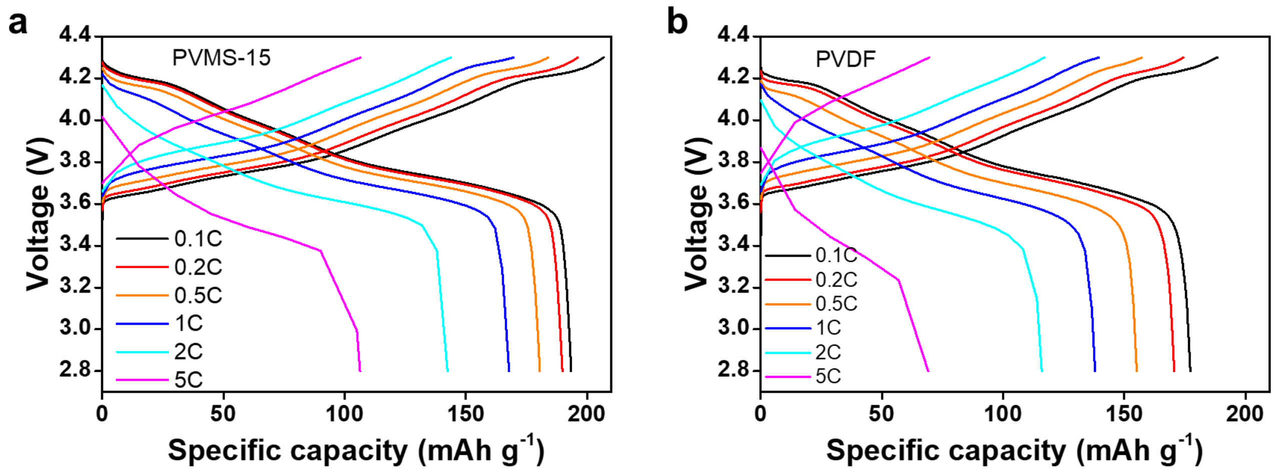

Supplementary Figure 35. Charge/discharge curves at different rates of Li|PVMS-15|NCM811 (a) and Li|PVDF|NCM811 (b) cells.

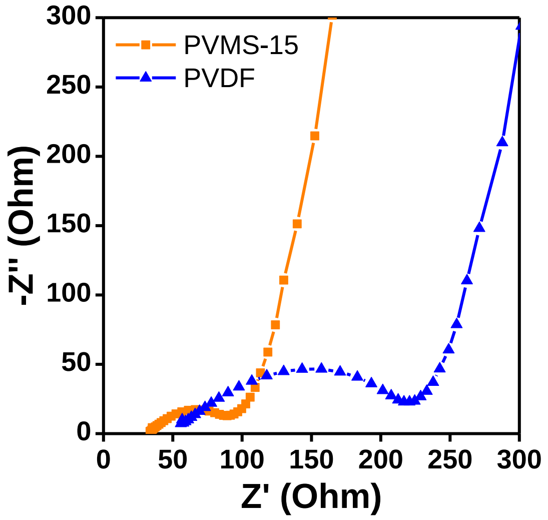

Supplementary Figure 36. EIS of Li|PVDF|NCM811 and Li|PVMS-15|NCM811 cells after rate tests.

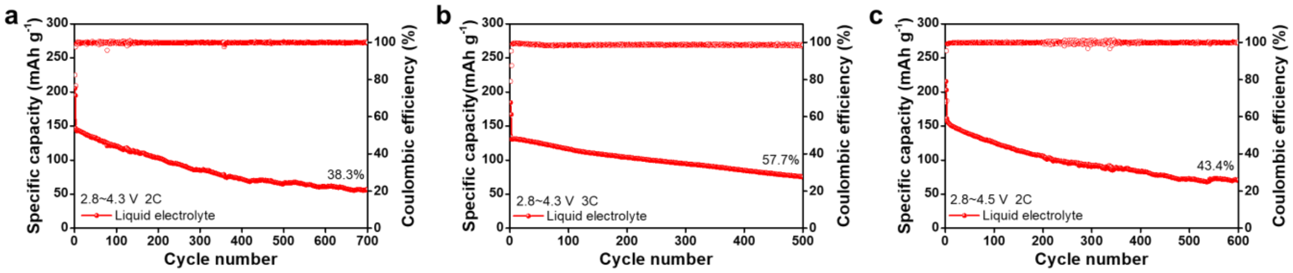

Supplementary Figure 37. Cycling stability of commercial liquid Li||NCM811 cells at 2.8~4.3 V, 2C (a), 2.8~4.3 V, 3C (b) and 2.8~4.5V, 2C (c).

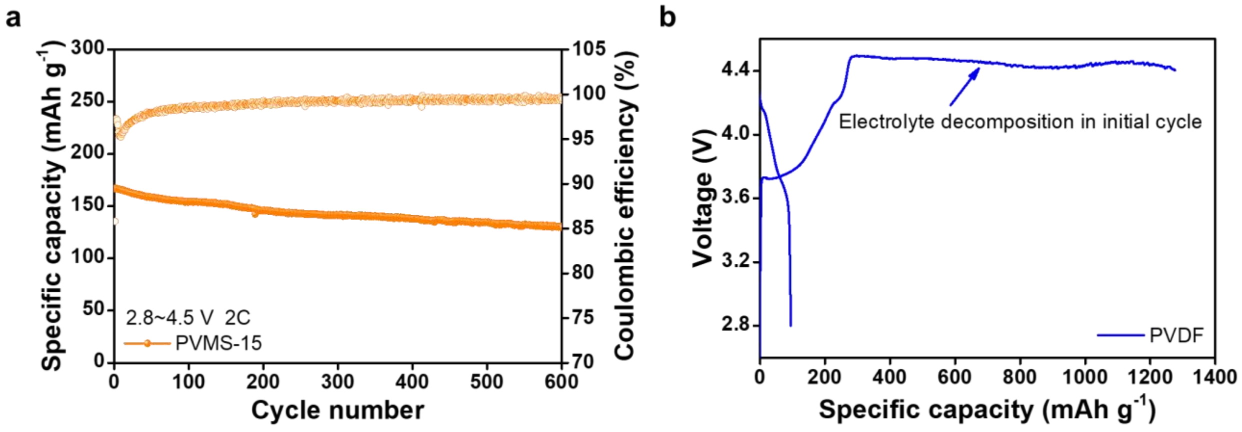

Supplementary Figure 38. Electrochemical performance of the batteries under cut-off voltage of 4.5 V. a Cycling stability of the Li|PVMS-15|NCM811 cells. **b** Charge/discharge curve of the Li|PVDF|NCM811 cells.

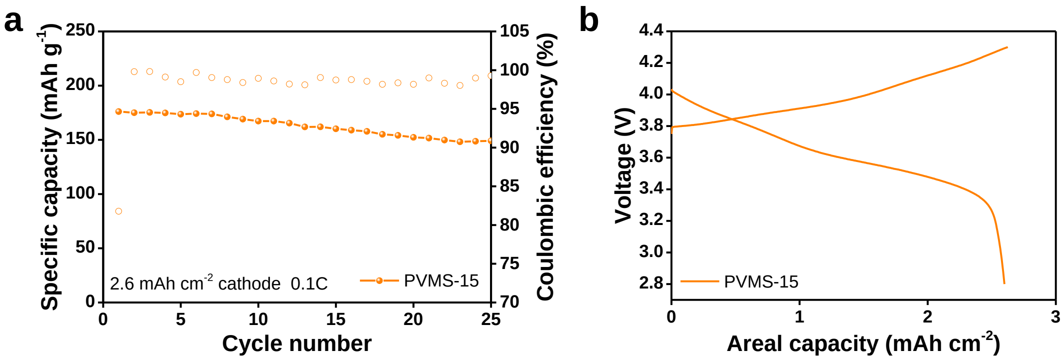

Supplementary Figure 39. Electrochemical performance of the Li||NCM811 coin cells with 2.6 mAh cm^{-2} cathode at 0.1C. a Cycling stability. **b** Charge/discharge curve.

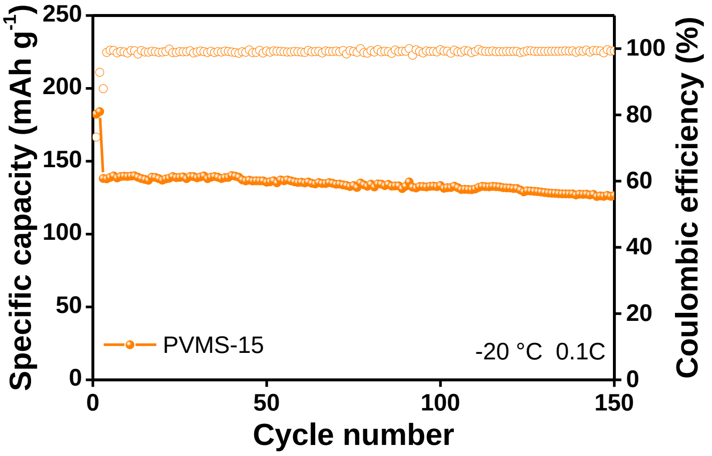

Supplementary Figure 40. Cycling stability of the Li|PVMS-15|NCM811 full cells at -20°C.

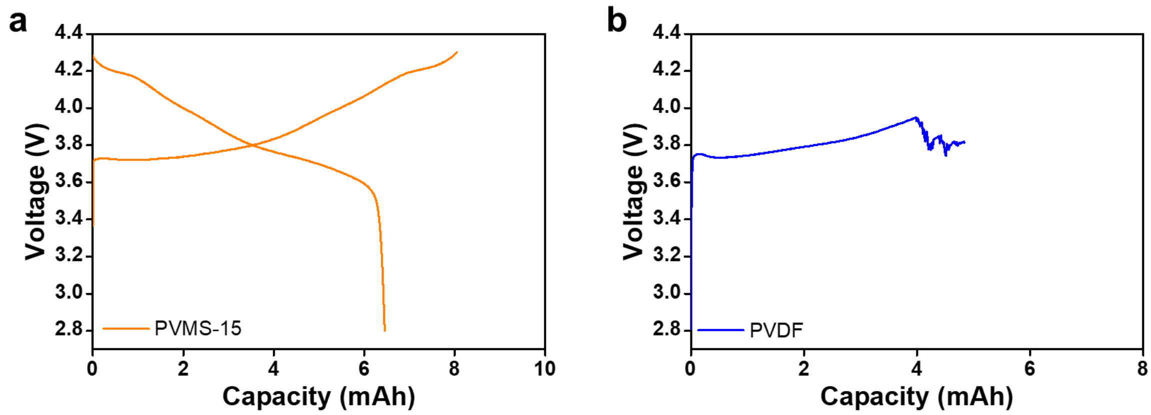

Supplementary Figure 41. Electrochemical tests of single layer pouch cells. Charge/discharge curve of Li|PVMS-15|NCM811 (**a**) and Li|PVDF|NCM811 (**b**) pouch cell.

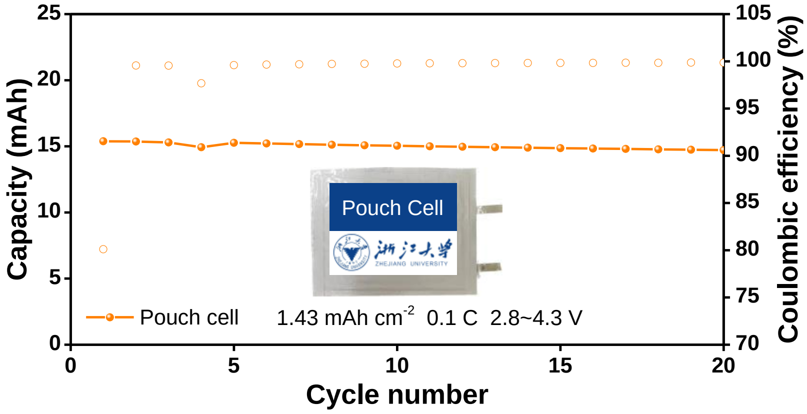

Supplementary Figure 42. Cycling stability of the Li|PVMS-15|NCM811 solid-state pouch cells.

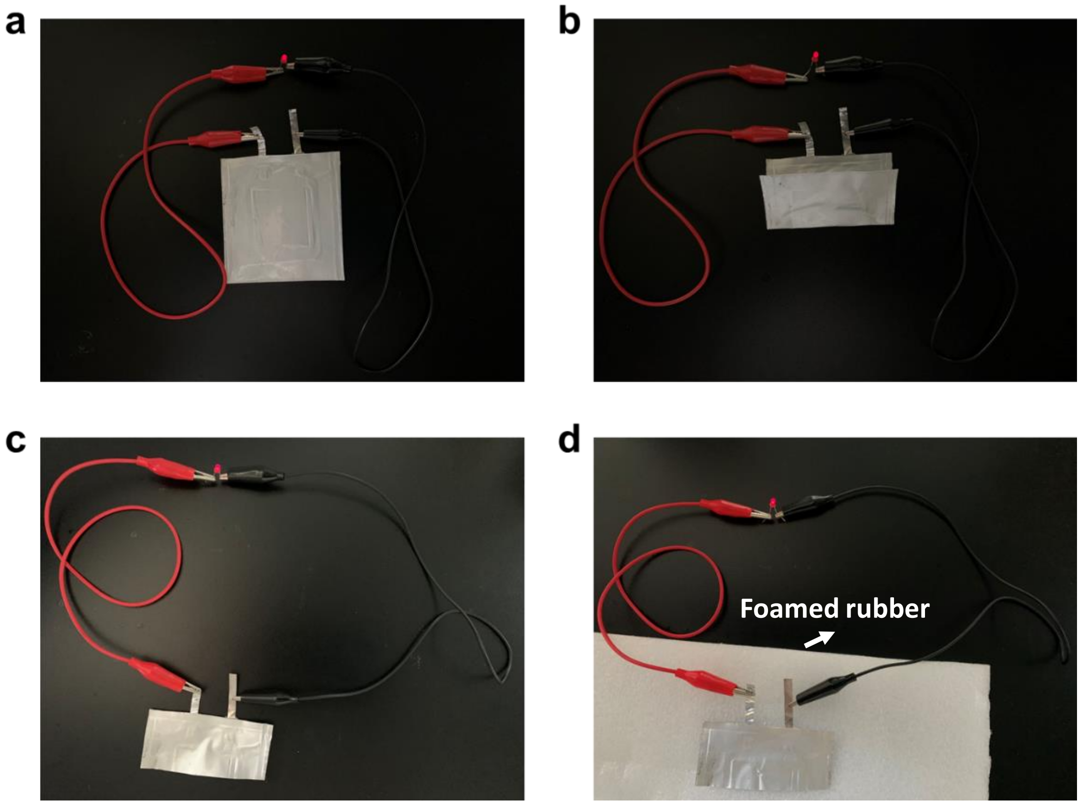

Supplementary Figure 43. Optical photographs of pouch cells using PVMS-15 electrolyte. a Optical photograph of Li|PVMS-15|NCM811 pouch cell. **b** Flexible test. **c** Cutting test. **d** Puncturing test.

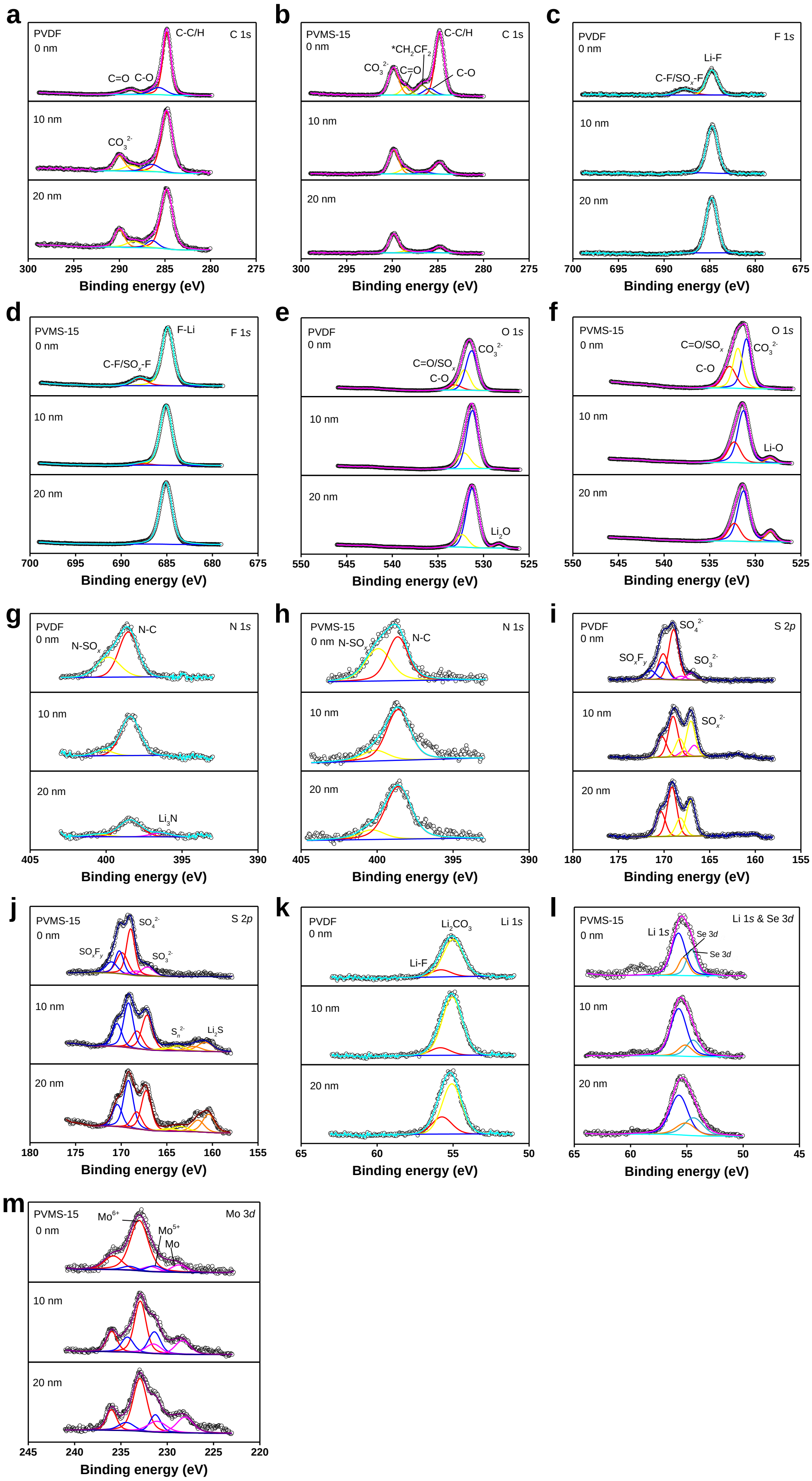

Supplementary Figure 44. XPS spectra of C 1*s* (a, b), F 1*s* (c, d), O 1*s* (e, f), N 1*s* (g, h), S 2*p* (i, j), Li 1*s* (k), Li 1*s* & Se 3*d* (l) and Mo 3*d* (m) for the SEI using PVDF and PVMS-15 electrolytes.

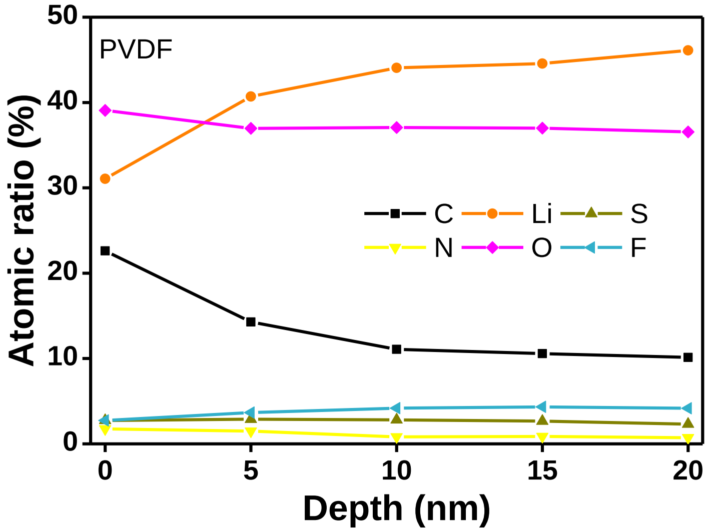

Supplementary Figure 45. Atomic ratios of the SEI at different sputter time with the PVDF electrolyte.

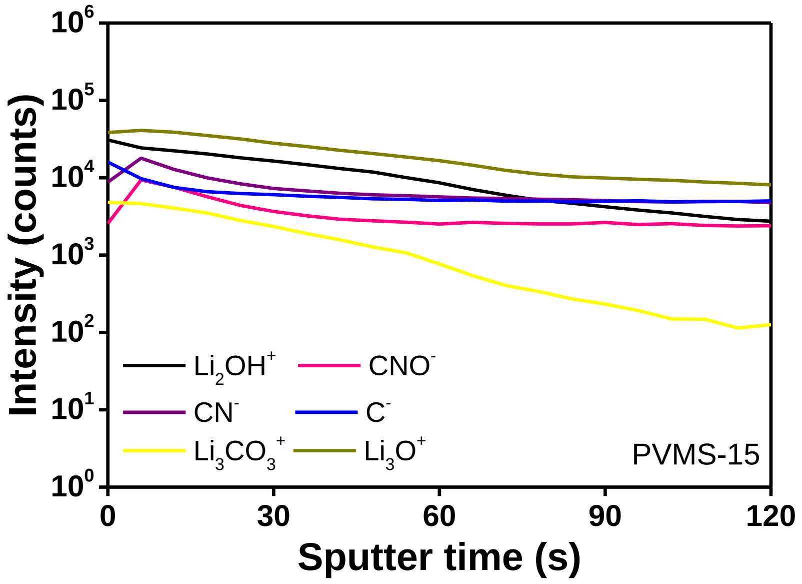

Supplementary Figure 46. TOF-SIMS depth profiles of secondary ion fragments in the formed SEI by PVMS-15 electrolyte.

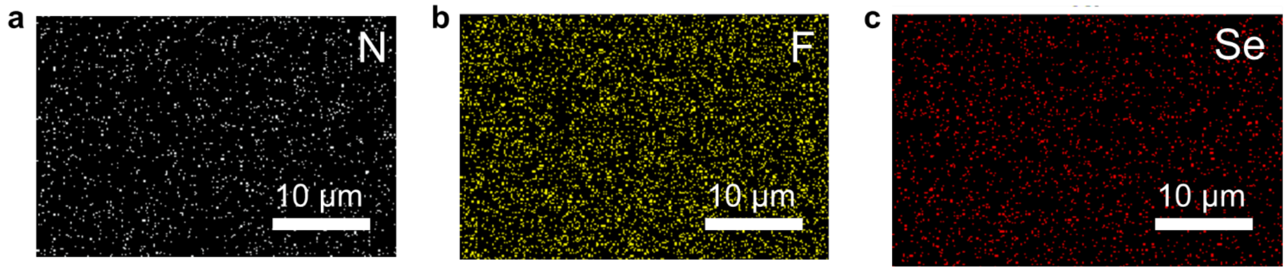

Supplementary Figure 47. Element EDS mappings of N (a), F (b) and Se (c) of SEI formed by the PVMS-15 electrolyte.

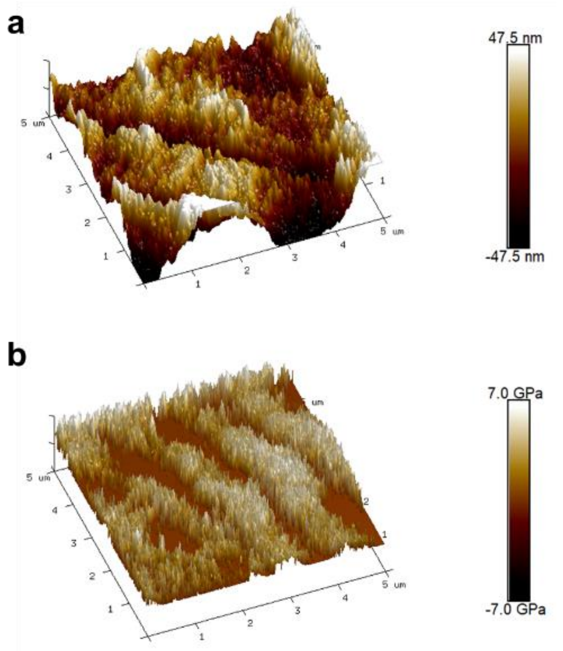

Supplementary Figure 48. 3D AFM images of roughness (a) and Young's modulus (b) tests of the SEI using the PVDF electrolyte.

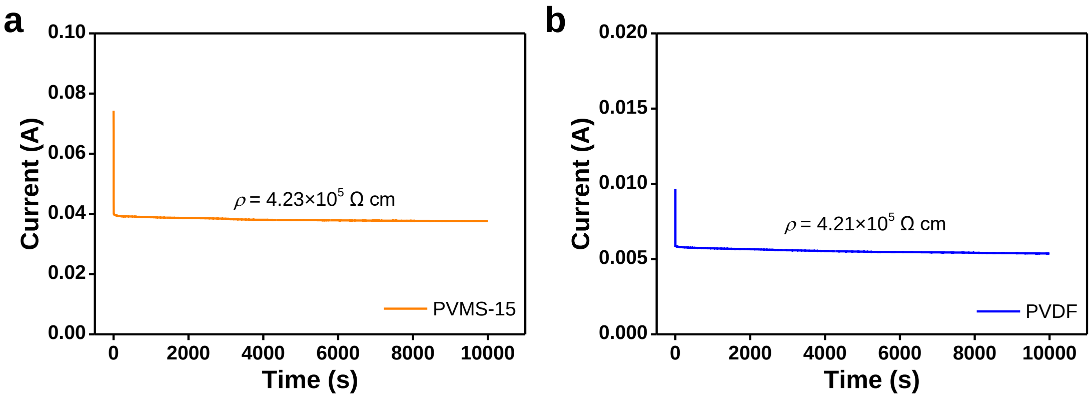

Supplementary Figure 49. Current-time curves of the Li|SEI|SS cells for the electronic conductivity of the SEI in PVMS-15 (a) and PVDF (b) electrolytes.

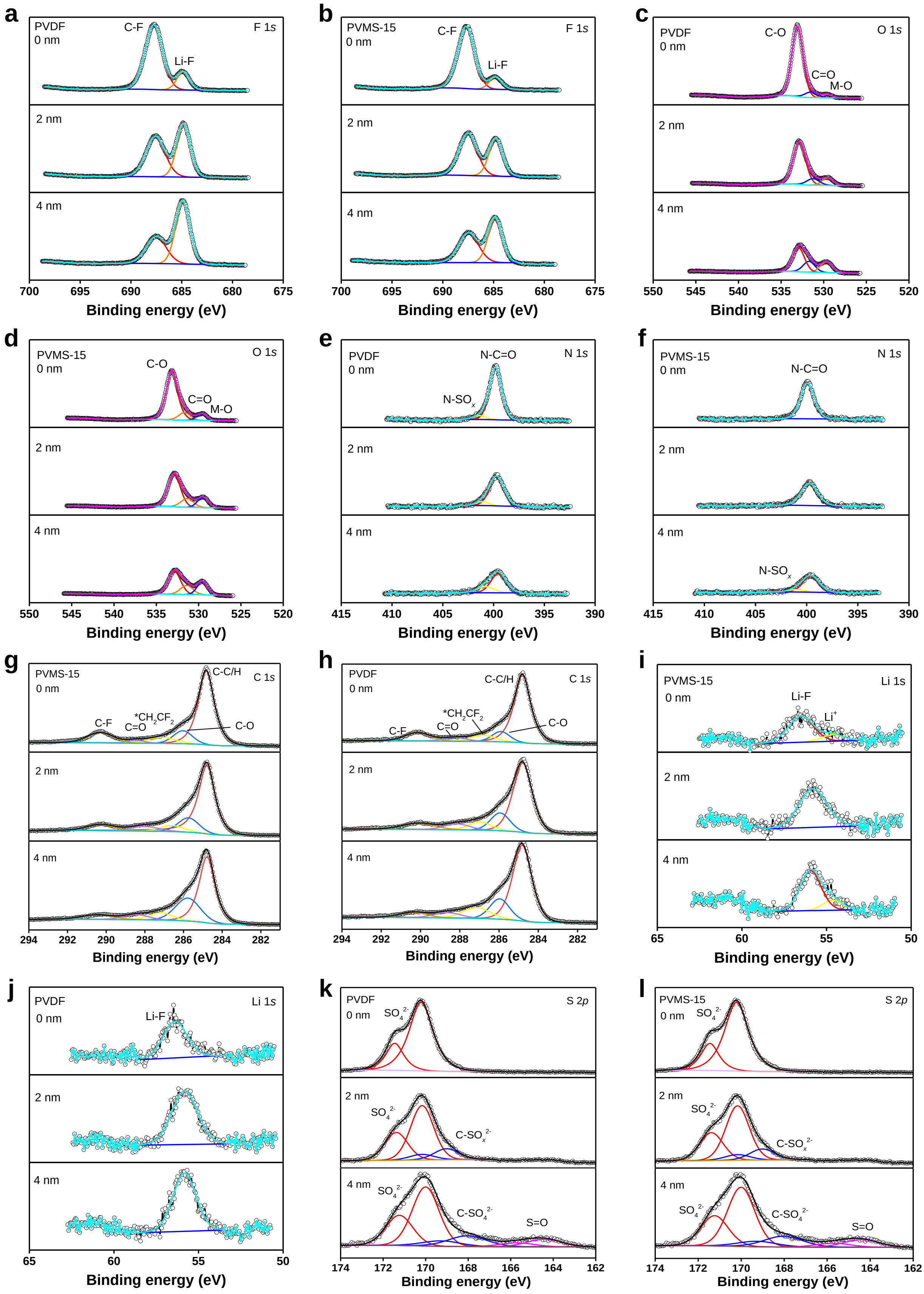

Supplementary Figure 50. XPS spectra of F 1*s* (a, b), O 1*s* (c, d), N 1*s* (e, f), C 1*s* (g, h), Li 1*s* (i, j) and S 2*p* (k, l) for the CEI using PVDF and PVMS-15 electrolytes.

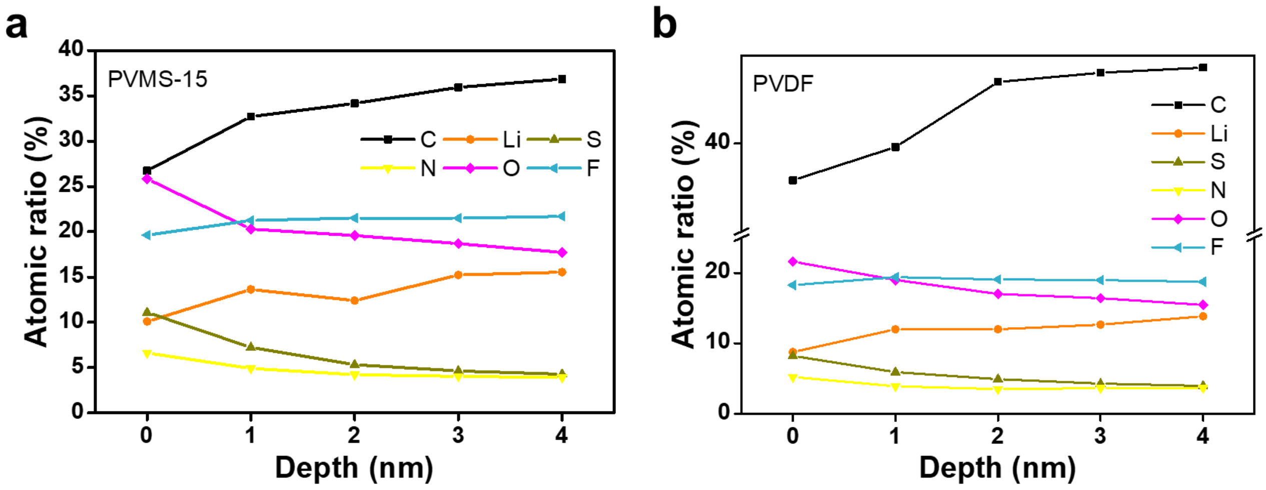

Supplementary Figure 51. Atomic ratios of the CEI at different sputter time with the PVMS-15 (a) and PVDF (b) electrolytes.

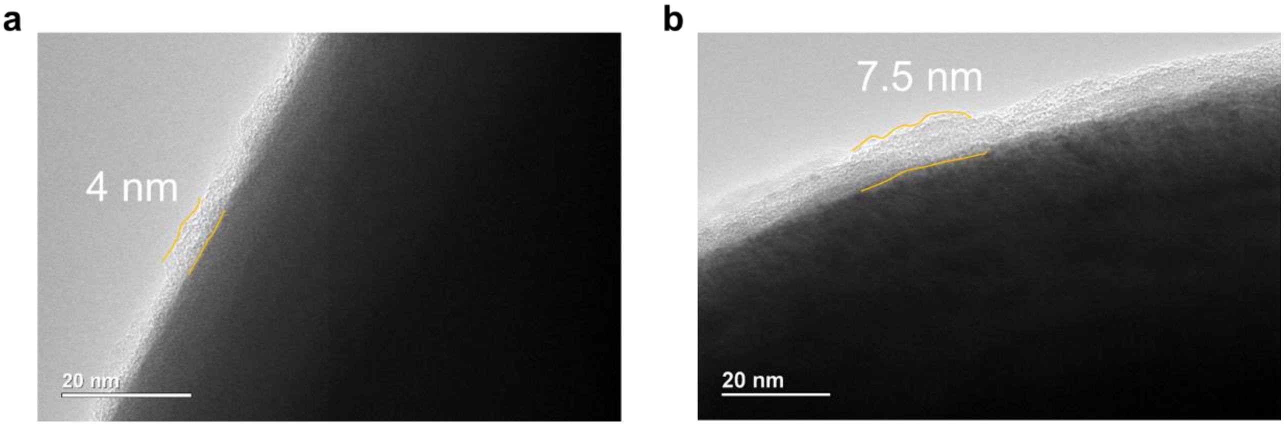

Supplementary Figure 52. CEI images. TEM images of the CEI using PVMS-15 (**a**) and PVDF (**b**) electrolytes.

Supplementary Tables

Supplementary Table 1. Calculation details of β-phase PVDF in the electrolytes.

Electrolyte	Average A_β	Average A_α	Average β-phase PVDF content (%)
PVDF	0.1598	0.1891	39
PVMS-10	0.2014	0.1430	52
PVMS-15	0.2422	0.1057	64
PVMS-20	0.2891	0.0661	77

Supplementary Table 2. Electronic conductivities of the electrolytes.

Electrolyte	Electronic conductivity (S cm^{-1})
PVDF	6.82×10^{-11}
PVMS-10	2.08×10^{-10}
PVMS-15	3.09×10^{-10}
PVMS-20	3.51×10^{-10}

Supplementary Table 3. Comparison of electronic conductivities of the PVMS-15 electrolyte with other reported electrolyte systems.

Electrolyte	Electronic conductivity (S cm^{-1})	Ref.
$Li_2In_{1/3}Sc_{1/3}Cl_4$	4.7×10^{-10}	8
Li_3YCl_6	2.8×10^{-9}	9
PEO/PVDF/LiTFSI /TiO_2	10^{-10}	10
LiTFSI/EmimFSI/ PMMA/LLZO	3.14×10^{-10}	11
Li_3PS_4	5×10^{-9}	12
LiF-$Li_{10}GeP_2S_{12}$	2.42×10^{-9}	13
$LiNbOCl_4$	5.22×10^{-10}	14
PEO/LiTFSI/GDC	5.5×10^{-10}	15
LLZO/Li_3AlF_6	1.27×10^{-8}	16
PI/LLZTO/PVDF	3.73×10^{-10}	17
PEO/LiTFSI/carbon	6.83×10^{-9}	18
PVMS-15	3.09×10^{-10}	This work

Supplementary Table 4. Calculated lattice parameters for Li_2Se, LiOH, Li_2CO_3, and Li_2O.

Crystals	Calculated	Reported	Space Group
Li_2Se	a= b=c=5.89 Å<br>α=β=γ=90°	a=b=6.02 Å<br>α=β=γ=90°	Fm3m (No. 225)
LiOH	a=b=3.51 Å c= 4.11 Å<br>α=β=γ=90°	a=b=3.59 Å c=4.41 Å<br>α=β=γ=90°	P4/nmm (No.129)
Li_2CO_3	a=8.37 Å b=5.02 Å c=6.32 Å<br>α=γ=90° β=114.08°	a=8.42 Å b=5.04 Å c=6.35 Å<br>α=γ=90° β=114.24°	C2/c (No. 15)
Li_2O	a= b=c=4.55 Å<br>α=β=γ=90°	a=b=c=4.66 Å<br>α=β=γ=90°	Fm3m (No. 225)

Supplementary Table 5. Calculated surface energies along different orientations for Li_2Se, LiOH, Li_2CO_3, and Li_2O.

Crystals	Surfaces	Surface energy (J m^{-2})
Li_2Se	002	2.07
	111	0.64
LiOH	001	0.25
Li_2CO_3	002	0.17
Li_2O	111	0.99
	110	1.16

Supplementary Table 6. Comparison of our developed batteries with other reported batteries.

Electrolyte	Ionic conductivity ($\times10^{-4}$ S cm^{-1})	Areal capacity (mAh cm^{-2})	Current density (mA cm^{-2})	Thickness (μm)	Cathode material	Cut-off Voltage (V)	Cycle number	Ref.
PVDF/LLZTO	5	0.342	0.1	100	LCO	4.2	120	19
PVDF-*b*-PTFE/LLTO	1.38	0.45	0.225	61	NCM532	4.35	550	20
PVDF-HFP/LiTFSI	1.24	0.378	0.075	100	NCM532	4.3	200	21
PVDF-HFP/LiTFSI	2.7	0.36	0.36	——	NCM811	4.2	800	22
P(VDF-TrFE-CTFE)/LiTFSI	3.1	0.272	0.136	70	LFP	4.2	200	23
PVDF/BTO/LLTO	8	0.288	0.288	67	NCM811	4.3	1500	24
P(VDF-TrFE-CTFE)/(Pyr13-TFSI)	5.75	0.17	0.17	130	LFP	4.2	1000	25
PEO/SN/FEC	10.1	0.34	0.17	300~400	LFP	3.9	2000	26
IL/VEC/OFHDODA /LiTFSI	13.7	0.187	0.099	100~200	NCM532	4.5	200	27
COF/$LiClO_4$	1.2	0.17	0.17	80~100	LFP	4.2	750	28
COF/LiTFSI	1.65	0.425	0.1	32	LFP	3.8	130	29
PVMS-15	6.5	0.36	1.08	80	NCM811	4.3	2000	This work
PVMS-15	6.5	0.36	0.72	80	NCM811	4.5	600	This work
PVMS-15	6.5	1.44	0.288	80	NCM811	4.3	100	This work
PVMS-15	6.5	2.6	0.26	80	NCM811	4.3	25	This work

Supplementary references

1. Yang K*, et al.* Stable Interface Chemistry and Multiple Ion Transport of Composite Electrolyte Contribute to Ultra-long Cycling Solid-State $LiNi_{0.8}Co_{0.1}Mn_{0.1}O_2$/Lithium Metal Batteries. *Angew. Chem. Int. Ed.* **60**, 24668-24675 (2021).
2. Zhai P, Yang Z, Wei Y, Guo X, Gong Y. Two-Dimensional Fluorinated Graphene Reinforced Solid Polymer Electrolytes for High-Performance Solid-State Lithium Batteries. *Adv. Energy Mater.* **12**, 2200967 (2022).
3. Shen Z*, et al.* Tuning the Interfacial Electronic Conductivity by Artificial Electron Tunneling Barriers for Practical Lithium Metal Batteries. *Nano Lett.* **20**, 6606-6613 (2020).
4. He Y*, et al.* Origin of lithium whisker formation and growth under stress. *Nat. Nanotechnol.* **14**, 1042-1047 (2019).
5. Hebb MH. Electrical Conductibity of Silver Sulfide. *J. Chem. Phys.* **20**, 185-190 (1952).
6. Neudeck BJ, Weppner W. Li_9SiAlO_8: a Lithium Ion Electrolyte for Voltages Above 5.4 V. *J. Electrochem. Soc.* **143**, 2198-2203 (1996).
7. Liang X*, et al.* A facile surface chemistry route to a stabilized lithium metal anode. *Nat. Energy* **2**, 17119 (2017).
8. Zhou L*, et al.* High areal capacity, long cycle life 4 V ceramic all-solid-state Li-ion batteries enabled by chloride solid electrolytes. *Nat. Energy* **7**, 83-93 (2022).
9. Asano T, Sakai A, Ouchi S, Sakaida M, Miyazaki A, Hasegawa S. Solid Halide Electrolytes with High Lithium-Ion Conductivity for Application in 4 V Class Bulk-Type All-Solid-State Batteries. *Adv. Mater.* **30**, 1803075 (2018).
10. Zhang KL*, et al.* Understanding Enhanced Ionic Conductivity in Composite Solid-State Electrolyte in a Wide Frequency Range of 10^{-2} -10^{10} Hz. *Adv. Sci.* **9**, e2200213 (2022).
11. Zhai Y*, et al.* Enabling High-Voltage "Superconcentrated Ionogel-in-Ceramic" Hybrid Electrolyte with Ultrahigh Ionic Conductivity and Single Li^+-Ion Transference Number. *Adv. Mater.* **34**, 2205560 (2022).
12. Li X*, et al.* High-performance all-solid-state Li–Se batteries induced by sulfide electrolytes. *Energy Environ. Sci.* **11**, 2828-2832 (2018).
13. Jin Y*, et al.* Fluorinated $Li_{10}GeP_2S_{12}$ Enables Stable All-Solid-State Lithium Batteries. *Adv. Mater.* **35**, 2211047 (2023).
14. Tanaka Y, Ueno K, Mizuno K, Takeuchi K, Asano T, Sakai A. New Oxyhalide Solid Electrolytes with High Lithium Ionic Conductivity >10 mS cm^{-1} for All-Solid-State Batteries. *Angew. Chem.* **135**, e202217581 (2023).
15. Wu N*, et al.* Enhanced Surface Interactions Enable Fast Li^+ Conduction in Oxide/Polymer Composite Electrolyte. *Angew. Chem. Int. Ed.* **59**, 4131-4137 (2020).
16. Biao J*, et al.* Inhibiting Formation and Reduction of Li_2CO_3 to LiC_x at Grain Boundaries in Garnet Electrolytes to Prevent Li Penetration. *Adv. Mater.* **35**, 2208951 (2023).
17. Hu J, He P, Zhang B, Wang B, Fan L-Z. Porous film host-derived 3D composite polymer electrolyte for high-voltage solid state lithium batteries. *Energy Storage Mater.* **26**, 283-289 (2020).
18. Guo X, Ju Z, Qian X, Liu Y, Xu X, Yu G. A Stable Solid Polymer Electrolyte for Lithium Metal Battery with Electronically Conductive Fillers. *Angew. Chem. Int. Ed.* **62**, e202217538 (2023).
19. Zhang X*, et al.* Synergistic Coupling between $Li_{6.75}La_3Zr_{1.75}Ta_{0.25}O_{12}$ and Poly(vinylidene fluoride) Induces High Ionic Conductivity, Mechanical Strength, and Thermal Stability of Solid Composite Electrolytes. *J. Am. Chem. Soc.* **139**, 13779-13785 (2017).
20. Liu S, Zhao Y, Li X, Yu J, Yan J, Ding B. Solid-State Lithium Metal Batteries with Extended Cycling Enabled

by Dynamic Adaptive Solid-State Interfaces. *Adv. Mater.* **33**, e2008084 (2021).

21. Liu W*, et al.* Designing Polymer-in-Salt Electrolyte and Fully Infiltrated 3D Electrode for Integrated Solid-State Lithium Batteries. *Angew. Chem. Int. Ed.* **60**, 12931-12940 (2021).
22. Xu S*, et al.* Decoupling of ion pairing and ion conduction in ultrahigh-concentration electrolytes enables wide-temperature solid-state batteries. *Energy Environ. Sci.* **15**, 3379-3387 (2022).
23. Huang Y-F*, et al.* A relaxor ferroelectric polymer with an ultrahigh dielectric constant largely promotes the dissociation of lithium salts to achieve high ionic conductivity. *Energy Environ. Sci.* **14**, 6021-6029 (2021).
24. Shi P*, et al.* A dielectric electrolyte composite with high lithium-ion conductivity for high-voltage solid-state lithium metal batteries. *Nat. Nanotechnol*. **18**, 602-680 (2023).
25. Liu J, Wu Z, Stadler FJ, Huang Y. High Dielectric Poly(vinylidene fluoride)-Based Polymer Enables Uniform Lithium-Ion Transport in Solid-State Ionogel Electrolytes. *Angew. Chem. Int. Ed*. **62**, e202300243 (2023).
26. Lin R*, et al.* Characterization of the structure and chemistry of the solid-electrolyte interface by cryo-EM leads to high-performance solid-state Li-metal batteries. *Nat. Nanotechnol.* **17**, 768-776 (2022).
27. Tang L*, et al.* Polyfluorinated crosslinker-based solid polymer electrolytes for long-cycling 4.5 V lithium metal batteries. *Nat. Commun.* **14**, 2301 (2023).
28. Li J*, et al.* Room temperature all-solid-state lithium batteries based on a soluble organic cage ionic conductor. *Nat. Commun.* **13**, 2031 (2022).
29. Guo D*, et al.* Foldable Solid-State Batteries Enabled by Electrolyte Mediation in Covalent Organic Frameworks. *Adv. Mater*. **34**, e2201410 (2022).
